# Genetic Structure and Evolution of the *Leishmania* Genus in Africa and Eurasia: What Does MLSA Tell Us

**DOI:** 10.1371/journal.pntd.0002255

**Published:** 2013-06-13

**Authors:** Fouad El Baidouri, Laure Diancourt, Vincent Berry, François Chevenet, Francine Pratlong, Pierre Marty, Christophe Ravel

**Affiliations:** 1 Department of Parasitology, Montpellier University Hospital, Montpellier, France; 2 Pasteur Institute, Genotyping of Pathogens and Public Health, Paris, France; 3 Méthodes et Algorithmes pour la Bioinformatique, LIRMM, UMR 5506 CNRS – Université Montpellier 2, Montpellier, France, Institut de Biologie Computationnelle, Montpellier, France; 4 MIVEGEC, CNRS 5290, IRD 224, Universités Montpellier 1 et 2, Montpellier, France; 5 Parasitologie-Mycologie, Centre Hospitalier Universitaire de Nice et Faculté de Médecine, Université de Nice-Sophia Antipolis, Inserm U 1065, Nice, France; Charité University Medicine Berlin, Germany

## Abstract

Leishmaniasis is a complex parasitic disease from a taxonomic, clinical and epidemiological point of view. The role of genetic exchanges has been questioned for over twenty years and their recent experimental demonstration along with the identification of interspecific hybrids *in natura* has revived this debate. After arguing that genetic exchanges were exceptional and did not contribute to *Leishmania* evolution, it is currently proposed that interspecific exchanges could be a major driving force for rapid adaptation to new reservoirs and vectors, expansion into new parasitic cycles and adaptation to new life conditions.

To assess the existence of gene flows between species during evolution we used MLSA-based (MultiLocus Sequence Analysis) approach to analyze 222 *Leishmania* strains from Africa and Eurasia to accurately represent the genetic diversity of this genus. We observed a remarkable congruence of the phylogenetic signal and identified seven genetic clusters that include mainly independent lineages which are accumulating divergences without any sign of recent interspecific recombination. From a taxonomic point of view, the strong genetic structuration of the different species does not question the current classification, except for species that cause visceral forms of leishmaniasis (*L. donovani*, *L. infantum* and *L. archibaldi*). Although these taxa cause specific clinical forms of the disease and are maintained through different parasitic cycles, they are not clearly distinct and form a continuum, in line with the concept of species complex already suggested for this group thirty years ago. These results should have practical consequences concerning the molecular identification of parasites and the subsequent therapeutic management of the disease.

## Author Summary

The mechanisms of genomic and genetic evolution in the *Leishmania* order, a protozoan group that contains about twenty pathogenic species, are the focus of much debate. Although these parasites have been considered for years to reproduce clonally, recent works have demonstrated both experimental and *in natura* intra- and inter-specific hybrids. Interspecific exchanges should be sources of plasticity and adaptation to new parasitic cycles. In this work we used a MultiLocus Sequence Analysis approach to analyze 222 *Leishmania* strains that belong to different species and were isolated in African and Eurasian foci. This analysis classified the different strains in seven robust genetic clusters that showed remarkable congruence of the phylogenetic message between them. From a taxonomic point of view, the seven clusters overlapped with most of the biochemical taxonomic groups currently in use except for species causing visceral forms of leishmaniasis. Contrary to what expected, we did not detect traces of interspecific recombination and genetic exchanges between the different species. Finally, these results should have consequences on the taxonomy, on our understanding of the epidemiology and on the therapeutic management of these infections.

## Introduction

Protozoa of the *Leishmania* genus are part of the Trypanosomatids family, which also includes American and African Trypanosomes that cause Chagas disease and sleeping sickness. Compared to other human vector-borne protozoan parasitic diseases, such as malaria or trypanosomiases, leishmaniasis appears to be a complex parasitic disease not only from a clinical, but also from an epidemiological and taxonomic point of view.

Leishmaniasis is endemic in 98 countries with 2 million cases reported each year, especially in the poorest regions. It is a polymorphic disease that can cause skin or mucosal injuries, or affect macrophages of the whole reticulo-endothelial system. This disseminated form (visceral leishmaniasis) is lethal if untreated [1]. Disease progression and therapeutic management depend not only on the host immunogenetic characteristics, but also on the parasitic species. Indeed, about 20 *Leishmania* species have been described worldwide as pathogenic for humans [2]. However, their identification can be ambiguous and controversial, thus complicating the therapeutic management of these affections.

The epidemiology of leishmaniasis is also complex. Parasites can be transmitted through different zoonotic and anthropo-zoonotic cycles that involve domestic and wild mammalian reservoirs which belong to nine different orders (rodents, canines, toothless mammals, marsupials…) [3]. *Leishmania* parasites are transmitted to mammalian reservoirs by blood-sucking Diptera belonging to the genera *Phlebotomus* and *Lutzomyia*. At least 93 sandfly species are proven or probable *Leishmania* vectors worldwide [2]. Due to this diversity of reservoirs and potential vectors and the various host and vector-parasite specificities, many cycles probably remain to be identified.

Finally, the taxonomy of the species belonging to the *Leishmania* genus is still debated [4], [5]. The division of the *Leishmania* genus in two sub-genera (i.e., *Leishmania* and *Viannia*), which was originally based on the parasite position in the insect digestive tract, has been confirmed by all subsequent studies. Similarly, the definition of the taxonomic groups, which has been mainly based on the species isoenzymatic characteristics since the 80's, is generally in line with the epidemiological and clinical data. However, the increasing number of strains analyzed and the development of molecular techniques have led to question the identification of some groups as species (e.g., *L. archibaldi*). Moreover, a genetic continuum between some groups has been highlighted, leading some authors to suggest a reduction in the taxa number [5].

In addition, although the possibility of cell fusion between different *Leishmania* strains in the insect vector has been experimentally demonstrated, questions remain on their frequency *in natura*, the possibility of interspecific hybridizations, their impact on genomic evolution and the existence of gametes with meiotic reduction [6], [7].

Although sequencing projects of entire genome of different *Leishmania* species will probably see the light in the coming years, only the genome of six species has been sequenced and made public so far [8]–[12]. Moreover, with the exception of a recent study on American *Leishmania* species, previous molecular analyses suffered from heterogeneity and usually concerned only one gene and a limited number of strains [13], [ review in 14].

Therefore, we developed a MultiLocus Sequence Analysis (MLSA)-based approach to analyze systematically several genes in 222 *Leishmania* strains from Africa and Eurasia that should accurately represent the genetic diversity of this genus. The obtained data might help improving our knowledge on the genetic structuration and genomic evolution mechanisms of this genus. Practically, it should also facilitate the molecular identification of *Leishmania* strains and thus improve the therapeutic care and epidemiological understanding of this disease.

## Materials and Methods

### Selection of loci and strains

Initially, 40 coding DNA sequences (CDS) that correspond to housekeeping genes and are evenly spaced in the *Leishmania* genome were investigated. To identify only single copy genes, a systematic Blast analysis was performed against three complete *Leishmania* genomes deposited in GenBank (*L. infantum*, *L. major* and *L. braziliensis*) [9]. Then, the nucleotide sequences of these three species were compared to map polymorphic and conserved regions within each gene and to eliminate CDS containing indels. Finally, seven loci that are located in the central or telomeric region of six different chromosomes and are considered as independent genetic units were selected. To investigate a possible genetic linkage between loci located on the same chromosome, the loci 31.0280 and 31.2610 (separated by 1.2 Mb) on chromosome XXXI (2.7 Mb) were chosen. The biological function of the housekeeping genes analyzed was not considered as a criterion for locus selection.

In all, 222 *Leishmania* strains isolated in Eurasia and Africa were analyzed. These strains were selected among 6,000 *Leishmania* strains deposited in the collection of the French Reference Centre on Leishmaniasis (Montpellier, France). They were mainly isolated from infected patients (n = 176, 79.3%), but also from mammalian reservoirs (n = 38, 17.1%) and insect vectors (n = 8, 3.6%) (Table S1). All samples taken from humans were anonymized. To be representative of the *Leishmania* genetic diversity, strains were selected on the basis of isoenzymatic and geographic criteria. For each strain, isoenzymatic data for 15 enzymatic systems were available and each zymodeme was represented by at least one strain. When a zymodeme was present in different countries, a strain from each of these countries was selected, if possible. The 222 strains originated from 43 countries and were representative of 110 zymodemes. According to the isoenzymatic-based taxonomy [15], these strains were representative of the 10 different *Leishmania* species currently described in Eurasia and Africa: *L. infantum* (n = 90), *L. major* (n = 42) *L. donovani* (n = 29), *L. tropica* (n = 18) *L. aethiopica* (n = 18), *L. archibaldi* (n = 9), *L. turanica* (n = 8), *L. gerbilli* (n = 4), *L. killicki* (n = 3) and *L. arabica* (n = 1). Although recent works have suggested the possibility of genetic exchanges in the *Leishmania* genus, including inter-specific hybrids, such hybrid strains were not included in our dataset [7], [16]–[24].

### Nucleotide polymorphism analysis

In all, 1,554 double-strand sequences were aligned and visually checked using the CodonCode aligner software v.4.0.4 (CodonCode Co., USA). Sequences were put in phase with the open reading frame. The locus size ranged from 486 to 810 bp and the concatenated sequence was 4,677 bp-long (Table 1). The 1554 sequences were deposited in GenBank under the following numbers: KC158588-KC160141. As *Leishmania* is mainly considered to be a diploid organism, a special attention was paid to the heterozygous positions [10]. Chromatograms were examined visually in both directions and usually results were easily interpreted as heterozygous when two peaks in a chromatogram overlapped. No tri-allelic site was found. Only one strain (L3538) was cloned to look for multi-clonal populations of parasites and the allelic profiles of the clones were identical.

**Table 1:** Analyzed genes and genetic diversity.

Locus	Gene	Size (bp)	No of LST	No of variable sites	No of singl.	No of PIS	π	Hd	G+C	No of heterozygous sites	dN/dS
**03.0980**	Elongation initiation factor 2 alpha subunit. Putative	678	37	84	17	67	0.029	0.86	0.56	9	0.015
**04.0580**	Spermidine synthase 1. Putative	711	35	81	10	71	0.028	0.85	0.61	8	0.114
**10.0560**	Zinc binding dehydrogenase-like protein	636	43	60	19	41	0.014	0.91	0.63	14	0.091
**12.0010**	Translation initiation factor alpha subunit. Putative	714	35	99	24	75	0.030	0.92	0.53	9	0.032
**14.0130**	Nucleoside hydrolase-like protein	642	24	53	7	46	0.019	0.88	0.61	4	0.113
**31.0280**	Hypothetical protein. conserved	810	61	111	26	85	0.031	0.96	0.59	23	0.286
**31.2610**	RNA polymerase II largest subunit	486	26	34	7	27	0.019	0.82	0.60	11	0.025
**Concatenated**		4677	140	522	110	412	0.025	0.89	0.59	78	0.094

LST, *Leishmania* Sequence Type.

No of singl., number of singleton.

PIS, Parsimony Informative Sites.

Π, average number of nucleotide differences per site between any two randomly selected sequences.

Hd, Haplotype diversity.

G+C, GC content.

dN/dS, rate of substitution at non-silent sites (dN) per rate of substitution at silent sites (dS).

The MEGA version4 program was used to calculate the number of variable nucleotide sites, the nucleotide diversity (average number of nucleotide differences per site between any two randomly selected sequences) and the transition/transversion ratio (R). Haplotype diversity (Hd) was calculated using the DnaSP software, version5 [25].

The possible selection pressure on these protein-coding sequences was checked using the dN/dS ratio test and the Z-test of selection based on the Nei-Gojobori method (implemented in the DnaSP software, version5, and in the MEGA 4.0 package, respectively) [26].

### Phylogenetic analysis and congruence test

Both the individual gene sequences and the concatenated sequences were analyzed. In each case, nucleotides were duplicated to avoid information loss due to ambiguous states (e.g., A to AA or Y to CT). To take into account the possible occurrence of genetic exchanges in our dataset, MLSA data were first analyzed using a network representation with the aim of replacing the bifurcating tree model with a “reticulating tree” model, in which the reticulations represent possible evolutionary processes other than lineal descent with modifications, such as horizontal gene transfers [27]. Maximum Likelihood (ML) trees were constructed using PhyML, version 3.0 [28], [29]. The best-fitting model for nucleotide substitution was identified using the Corrected Akaike information Criterion (AICc) and Bayesian Information Criterion (BIC) implemented in JModelTest [28], [30]. The General Time-Reversible model was chosen with a proportion of invariables sites (I) and gamma-distributed (G) rate variation across sites (i.e., GTR+I+G). For the MultiLocus Enzyme Electrophoresis (MLEE) data analysis, isoenzyme data were transformed to produce a binary matrix (presence/absence of a band with a given mobility). Based on the hypothesis that *Leishmania* parasites are ‘mainly’ diploid, multiband patterns in starch gels were considered to be heterozygous and the electromorph values were duplicated. The Nei's index was calculated with the PhylTools package, version 1.32, to construct a distance matrix [31], [32]. This distance was preferred to other distance measures because it does not use the shared absence of a given allele as common characteristic [33]. Bootstrap values were collected from 1,000 replications of the bootstrap procedure using PylTools. The Neighbor and Consense programs of the Phylip package, version 3.6, were used to obtain the final MLEE Neighbor Joining (NJ) tree [34]. To compare the MLSA and MLEE tree topologies, a NJ tree for the MLSA data was generated in MEGA, version4, by using the Jukes-Cantor model and the same data set (222 strains, 110 zymodemes) [26]. Split decomposition and Neighbor-Net (NN) analyses were performed with SplitsTree, version4.11.3, by using p-distances and equal edge lengths [35]. The analysis was performed using both ambiguous nucleotide sites and duplicated nucleotide sites. All these phylogenetic analyses were done with 1,000 bootstrap (BP) replicates. Three species from South America belonging to the *Viannia* subgenus were used as an out-group for the ML phylogenetic analysis (Table S1). These distantly related strains were selected to prevent derived characters to be wrongly considered as common ancestral characters.

Non-parametric Shimodaira-Hasegawa (SH) tests implemented in the PAUP*4.0b10 package were used to test the tree topology congruence [36]. For a given dataset, the SH test uses the difference in log likelihoods of competing topologies as the test statistic. The null distribution of the test statistic (differences in log likelihoods) was obtained by using 1,000 replicates of non-parametric bootstrapping of re-estimated log likelihoods. To avoid potential bias toward higher levels of significance due to small numbers of topologies, 100 random topologies where added to each test.

Tree congruence was also assessed by using the topological supertree approach (PhySIC_IST) [37]. This non-plenary supertree, which does not necessarily contain all the taxa present in the source trees, discarding those position which greatly differed among the source trees or for which insufficient information was provided [37]. The informativeness of a supertree is estimated using a variation of the CIC (Cladistic Information Content) criterion that takes into account both the presence of multifurcations and the absence of some taxa. This is basically proportional to the number of complete binary trees that are compatible with the evaluated supertree. The Le and Gascuel (LG) replacement matrix that incorporates the rate variability across sites in likelihood calculations and the replacement rate estimations (implemented in PhyML, version 3.0) was used with a proportion of invariables sites (I) and gamma-distributed (G) rate variations across sites (*i.e.*, LG+I+G) to infer the best amino acid replacement matrices for ML tree topology [38].

### Recombination detection

Evidence for recombination between different genotypes was assessed using several methods (pairwise homoplasy index, substitution distribution methods and phylogenetic methods) as analyses of simulated data showed that no single method is optimal, whereas multiple approaches may maximize the chances of detecting recombination events [39]. The pairwise homoplasy index (PHI test) implemented in SplitsTree, version 4.11.3, calculates the mean of the refined incompatibility scores (representing the minimum number of homoplasies that have occurred in the history of the aligned sequences between two sites) obtained for nearby nucleotide sites along the sequences. Normal approximation of a permutation test was used to assess the significance of the PHI statistic for the presence of recombination (p-values<0.05 indicate significant presence of recombination) [40].

Substitution/distribution-based methods (GENCONV, MaxChi and the 3Seq algorithms implemented in the RDP3 package) test for significant clustering of substitutions within gene sequences, while phylogenetic methods (RDP algorithm implemented in the RDP3 package) search for significant variability in tree topologies among adjacent sequence fragments [41]–[45]. All these algorithms focus only on polymorphic sites within sequence triplets selected from a larger alignment. The major advantage of the substitution/distribution methods relative to pure phylogenetic and distance-based approaches is that they often allow the detection of recombination events that cannot, for example, be visualized as sequences “jumping” between clades of phylogenetic trees constructed using different alignment partitions [46]. Evidence for recombination was accepted if significant (p<0.01) and detected by at least two tests.

## Results

### Phylogenetic analysis of African and Eurasian *Leishmania* strains identifies seven main genetic clusters

In this study, 222 *Leishmania* strains isolated in Africa and Eurasia and that belong to 10 different *Leishmania* species (identified by the traditional biochemical criteria) were selected using both geographical and biochemical (isoenzymatic) criteria (see M&M section and Table S1). After a selection step (see M&M section), seven single copy coding DNA sequences located on six different chromosomes were amplified and sequenced, giving a 4,677 bp-long concatenated sequence (Table 1). In all, 522 polymorphic sites including 412 informative parsimonious sites and 110 singletons were identified. Depending on the locus analyzed, the number of segregating sites ranged from 34 (locus 31.2610) to 111 (locus 31.0280) and the percentage of polymorphic sites from 7% to 13.7% (mean = 11.2%) (Table 1). This polymorphism frequency was similar to the one reported for the *Leishmania Viannia* sub-genus (8.25%) and for *Trypanosoma cruzi* (6%) [13], [47]. The nucleotide diversity varied from π = 0.014 (locus 10.0560) to π = 0.031 (locus 31.0280) (mean value: π = 0.025) and the haplotype diversity ranged from 0.82 (locus 31.2610) to 0.96 (locus 31.0280). Overall, the number of genotypes per locus was between 24 and 61 (median value = 35, Table 1) and was similar to the results presented by Boité et al. on South American *Leishmania* strains belonging to the *Viannia* sub-genus (25 to 43, median value = 33) [13]. Statistical analysis of the selection pressures acting on the seven loci indicated that both the dN/dS ratio and the transition/transversion ratio (R) were strongly biased toward synonymous mutations (dN/dS ranged from 0.015 to 0.286 and R from 1.41 to 6.66; Table 1). This was probably due to the counter-selection of deleterious mutations during the evolution of these genes. Such a strong purifying selection was expected for housekeeping genes that ensure the proper working of the basic molecular machinery of life. This result was confirmed by the codon-based test of neutrality that rejected the null hypothesis of strict-neutrality (dN = dS) in favor of purifying selection (p<0.05) for the seven loci analyzed.

**Figure 1:**
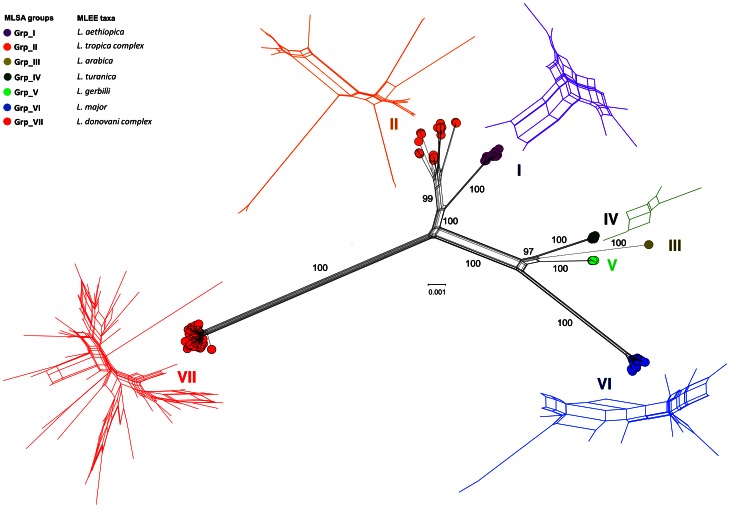
The Neighbor net analysis identifies seven distinct genetic clusters. Neighbor-Net analysis of the concatenated nucleotide sequences based on uncorrected p-distance matrices. Bootstrap values (1,000 replicates) are shown on the edges (percentages). Seven different genetic clusters were identified (I to VII). The MLEE-based taxa and MLSA-based clusters (I to VII) are color-coded. Sub-networks of clusters I, II, IV, VI and VII are magnified and color-coded. Clusters III and V are not magnified due to the low number of genotypes.

The systematic comparison of the concatenated sequences of the 222 strains gave rise to 140 different genotypes of which 124 were unique. Each genotype was identified by a LST prefix (*Leishmania* Sequence Type; see Table S1). Fifteen genotypes were identified in two to six strains (LST0002 to LST0016) and genotype LST0001 was the most common (52 strains) (Table S1), probably due to oversampling of this ubiquitous genotype in the Mediterranean basin. The unrooted Neighbor-Net (NN) network of the concatenated duplicated sequences was well resolved (least square fit 99.23%, Figure 1). The NN network analysis based on concatenated non-duplicated sequences (with ambiguous sites) gave identical results (Supplementary Figure S1). Although several conflicting signal patterns (box-like structures) were detected, the prevailing structure of the network was tree-like. Seven highly supported clusters (bootstrap percentage, BP = 99 to 100%) were clearly identified (I to VII, see Figure 1). The Split-decomposition analysis (fit index = 84.43%) gave a similar pattern with seven strongly supported clusters (BP = 98 to 100%, Figure S2). Box-like structures were less conspicuous than in the NN analysis.

Clusters I, III, IV, V and VI included strains belonging to the MLEE-based species *L. aethiopica*, *L. arabica*, *L. turanica*, *L. gerbilli* and *L. major*, respectively. Cluster II included the *L. tropica* and *L. killicki* strains and cluster VII was the largest and comprised strains from *L. donovani*, *L. infantum* and *L. archibaldi*. The precise demarcation of the species belonging to the seven clusters is discussed below. Although cluster III was represented by only one strain, this divergent genotype was considered as a distinct genetic entity and should be validated by additional strains analysis.

Then the validity of the seven clusters was confirmed by Maximum-likelihood (ML) and Bayesian probabilistic approaches that gave very similar topological patterns (Figure S3). The congruence between the two trees was assessed using the SH test. When tested against 100 random trees, the difference of likelihood between the Bayesian and the ML tree was negligible (−ln L = 51.25) in comparison to the difference between the random trees and the ML tree (−ln L = 44123 to 54958; p<0.05), indicating that the ML and Bayesian trees were significantly congruent (Table S2).

In both the ML and Bayesian trees, seven genetic clusters were clearly discernible and highly supported (BP = 100% and posterior probability, PP = 1) (Figure S3). These seven clusters were identical to those described in the network-based analysis, proving a phylogenetic signal agreement whatever the approach used.

### Locus contributions and inter-locus comparisons: a strong congruence

To assess the contribution of the seven loci to the main phylogenetic signal we compared the phylogenetic signal given by each locus to the overall signal using different methods. Overall, no major discrepancies were visually found in the seven individual ML trees (GTR+I+G model, Figure S4A). In these single-locus trees, most of the seven genetic clusters (I to VII) were easily identified and supported by BP (bootstrap percentage) above 98% (not shown). However, cluster II was not well supported in three of the seven tree loci (*i.e.*, 10.0560, 14.0130 and 31.2610) and clusters I and IV were weakly individualized in the analysis of locus 31.2610, probably due to a low number of parsimony informative sites (n = 27) (Figure S4A). The consistency of the tree congruence was statistically tested using the SH test. All seven loci were significantly congruent, except the locus 31.2610 topology that was rejected (p-value = 0.04) when compared to the data of locus 10.0560 (Table 2).

**Table 2:** Statistical assessment of congruence between the tree topologies of the seven housekeeping genes.

	03.0980	04.0580	10.0560	12.0010	14.0130	31.0280	31.2610
**03.0980**	(best)	0.34	0.10	0.19	0.21	0.09	0.27
**04.0580**	0.32	(best)	0.08	0.26	0.23	0.09	0.21
**10.0560**	0.31	0.29	(best)	0.10	0.13	0.07	0.23
**12.0010**	0.32	0.33	0.05	(best)	0.33	0.09	0.21
**14.0130**	0.31	0.30	0.05	0.21	(best)	0.10	0.21
**31.0280**	0.33	0.32	0.12	0.20	0.32	(best)	0.31
**31.2610**	0.25	0.14	0.04	0.06	0.12	0.09	(best)
**Concatenated**	0.60	0.58	0.31	0.67	0.63	0.57	0.64

P-value of the Log likelihood differences (pairwise comparisons performed using the SH test) for the seven loci analyzed. The only significant incongruence (p-value<0.05) is in bold. The highest p-values were noted “best”.

The good overall congruence was confirmed by PhySIC_IST, a topological approach that combines different rooted tree topologies in a supertree (Figure S4B) [37]. The resulting supertree did not contain relationships that contradicted the source trees. The supertree included all 140 genotypes and had a CIC value of 0.7 (*i.e.*, more than 70% of the supertree was resolved, indicating that the source trees were fairly congruent). However, the genotypes of clusters I and II were unresolved and appeared as a trifurcated branch. These two groups seemed to share common phylogenetic signals, suggesting possible ancestral genetic exchanges between them.

To compare the phylogenetic signal of both nucleotide and amino acid (translated) sequences we first used an LG amino acid substitution model to build an ML tree from the 1,559 concatenated residues, including 152 non-synonymous changes (Figure S5). The seven clusters (I to VII) were highly supported (BP up to 94%). Although the tree topologies of individual loci were apparently poorly resolved due to the low number of alleles (except for locus 31.0280), the main phylogenetic signal was clearly similar to the one deduced using nucleotide sequences. The topological congruence between these two trees was confirmed by the SH test (Table S3) against 100 random trees (−ln L = 941.24 *vs.* mean −ln L = 48971.54; p<0.05 for random tree topologies). After subtraction of the locus 31.0280 data, similar results were obtained, indicating that the prevailing phylogenetic signal was not due mainly to the contribution of this locus (data not shown).

### Recombination and mutation

**Figure 2:**
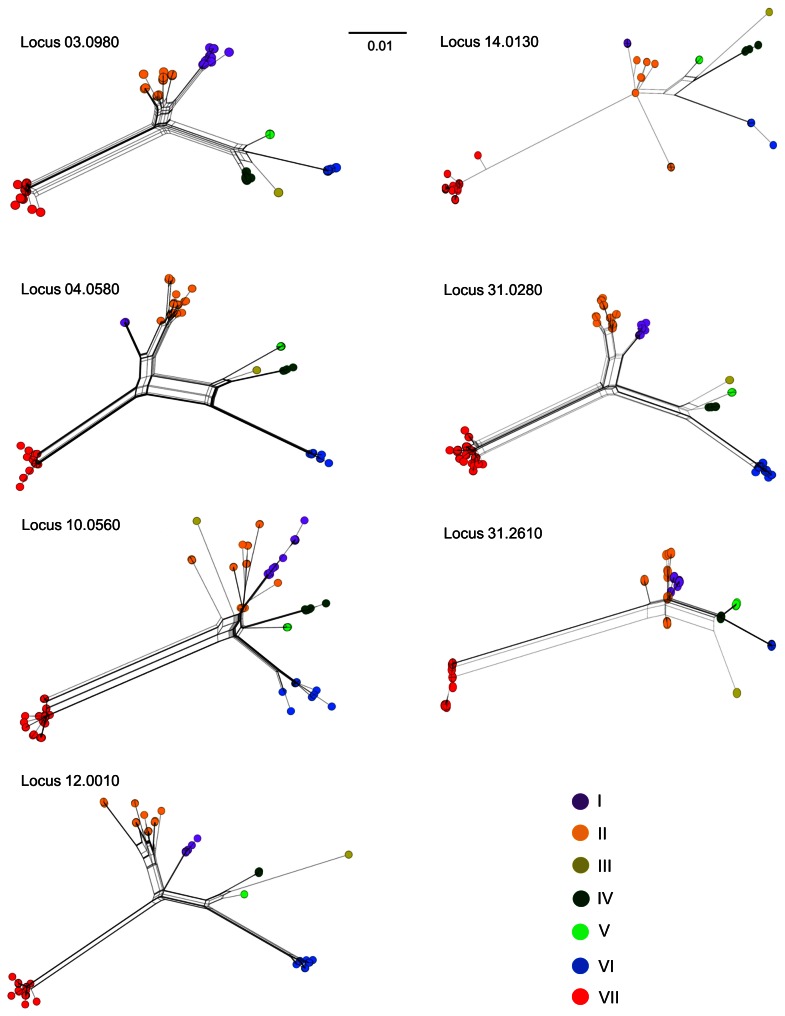
Individual Neighbor net analysis of the seven housekeeping genes. Neighbor-Net analysis of each of the seven gene sequences based on uncorrected p-distance matrices. The seven genetic clusters are color-coded according to Figure 1.

*Leishmania* is currently considered as diploid organism although the occurrence of aneuploid chromosomes could be a frequent event [10]. In the present study, heterozygous sites were detected at all the loci analyzed, confirming that the *Leishmania* genome is at least partially diploid. Across the 4,677 bp analyzed, heterozygous sites were found in 32.9% of the selected strains and characterized 1.67% of the polymorphic sites (11, 13, 19 and 30 strains were heterozygous for 4, 3, 2 and 1 polymorphic site, respectively). Heterozygous strains were representative of the different *Leishmania* species under study. Most of the heterozygous sites were CT or AG transitions (58% Y, 21% R, 9.5% S, 7% M, 2% W and 1.5% K). Locus 31.0280 contained the largest number of heterozygous sites, whereas locus 14.0130 the smallest (only 7% heterozygosity, Table 1). This value was slightly higher than the one reported by Boité et al. for strains of the *Leishmania Viannia* sub-genus, possibly due to the smaller number of strains analyzed [13]. To further investigate the possibility of genetic exchanges among the strains under study, the seven ML tree topologies were visually checked systematically. If separated phylogenetic trees are constructed using sequences corresponding to the two tracts of a recombinant sequence inherited from different parents, the recombinant sequences are expected to apparently jump between clusters when two trees are compared [46]. Among the seven clusters analyzed, only cluster II exhibited such topological rearrangement. Within this cluster some genetic sub-groups were well conserved across the different loci analyzed. Generally, these sub-clusters were constituted of strains coming from one or more neighboring countries (e.g., LST59/LST65/LST69 from Morocco, LST68/LST74/LST77 from Kenya, LST16/LST121 from Tunisia and the whole cluster I from Ethiopia and Kenya). However, some genotypes (LST0032, LST0070 and LST0115) grouped in cluster II on loci 03.0980, 14.0130, 31.0280 and 31.2610 were reshuffled on loci 04.0580, 10.0560 and 12.0010 (Figure S6). Similar clues of genotype recombination within cluster II were observed using the PHI-test after NN analysis of each individual locus (Figure 2) and of the concatenated data set [40].

Although the resulting networks exhibited a tree-like structure and were well resolved (least square fit >98.5%), significant PHI-test values in favor of recombination were detected for cluster II genotypes in locus 04.0580 (p = 0.023) and for cluster II and cluster VII in the concatenated data (p = 0.001 and p = 0.047, respectively).

Substitution/distribution-based methods implemented in RDP3 could not detect any intra-genic recombination (highest *p*-value = 0.01). When the seven loci were concatenated only one recombination signal involving three genotypes (LST0032, LST0045 and LST0115) was detected by two of the four tests (MaxChi and 3Seq, *p*-value = 0.01), although it was not possible to identify the parental genotypes. This result was congruent with the visual comparisons of the ML tree topologies described above for cluster II.

The analysis of the data for each cluster identified 78 heterozygous positions in total and more than half of them were in cluster II (n = 20) and VII (n = 22). Among these positions, 63% (n = 42) could be explained by recombination of parental alleles present in the same cluster. However, linkage analysis (RDP3, PHI-test) to test the possibility of recombination between neighboring alleles did not show any recombination signal, except between the genotypes LST0045, LST0032 and LST0115. Nevertheless, the signal was weak and was not possible to identify without ambiguity which of the three genotypes would result from the hybridization of the two others. Surprisingly, in 30 (38%) of the remaining heterozygous positions, the second parental allele was not found in the same cluster or in the other six clusters. Overall, of the 78 heterozygous positions identified in our dataset, 29 (37%) were orphan of one allele and therefore probably resulted from mutations that remained in the current populations rather than from recombination events.

### Comparing the MLSA- and MLEE-based clusters

**Figure 3:**
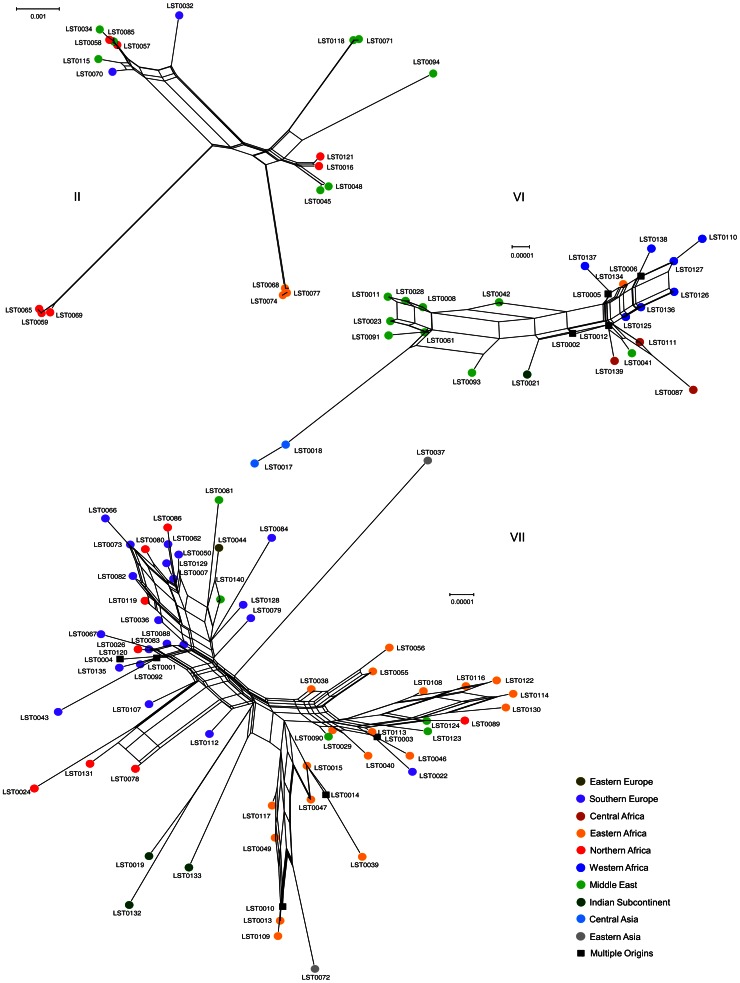
Geographical distribution of cluster II, VI and VII genotypes. The neighbor-Net analyses of clusters II, VI and VII (defined in Figure 1) are represented. The genotypes are color-coded according to their geographical origins (detailed in Table S1).

**Figure 4:**
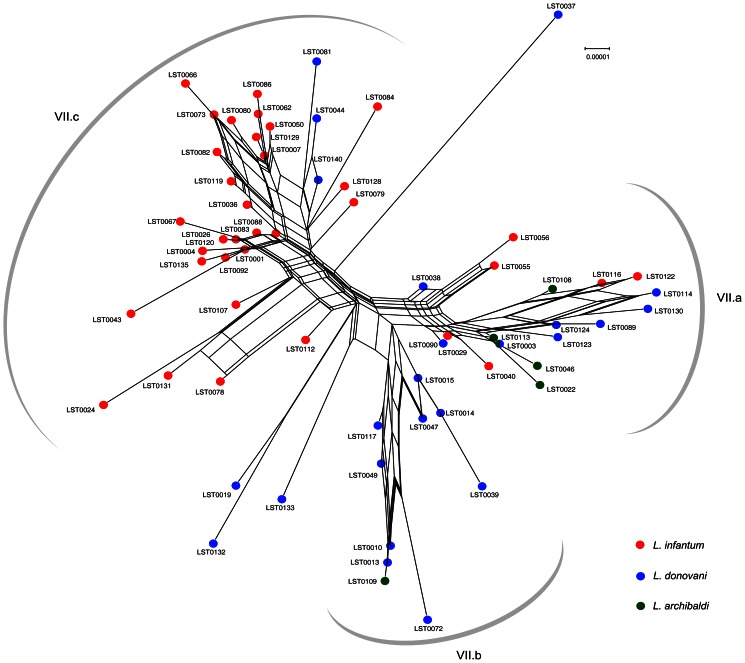
Merging the MLEE-based taxa and the MLSA-based cluster VII. Network representation of the Neighbor-Net analyses of cluster VII. The genotypes are color-coded according to the biochemical taxonomy of the strains belonging to this cluster. To facilitate the interpretation of Figure 4 three sub-networks (VIIa, VIIb and VIIc) are represented.

The 222 strains from Africa and Eurasia analyzed in this study belonged to 10 different *Leishmania* species, according to the current MLEE-based taxonomy. The major criterion for the *Leishmania* taxonomy has been based for up to 20 years on the systematic analysis of 13 to 15 isoenzymes [15]. However some recent studies based on different genetic markers have challenged the MLEE taxonomy and the species or sub-species status of some groups is currently under debate [4], [5]. Especially, systematic sequencing of allozyme coding genes revealed some genotype-phenotype discrepancies: for instance, indistinguishable phenotypes could be due to distinct genotypes and, conversely, identical genotypes could produce distinct phenotypes [48], [49]. To assess whether the MLEE phylogenetic signal (15 different enzymatic systems) and the MLSA phylogenetic signal of the seven loci under study were similar, we compared the MLSA and MLEE tree topologies of the 222 strains (Figure S7). For clusters I, II, IV, V, VI and VII, both the MLSA and the corresponding MLEE-based NJ trees were well supported (100% for all the MLSA clusters and 100%, 55%, 100%, 100%, 98% and 100% for the MLEE groups). Cluster III was represented by a single genotype and the BP calculation was not relevant. Overall, the main MLEE-based taxonomic groups were clearly confirmed by the SH test (p-value = 0.16, random trees *p*-value<0.05; Table S4). Cluster I to VII corresponded to the MLEE-based taxonomic groups *L. aethiopica*, *L. tropica* complex, *L. arabica*, *L. turanica*, *L. gerbilli*, *L. major* and *L. donovani* complex, respectively. Conversely, the intra-cluster supports were significantly higher for MLSA than for MLEE (median BP from 37.5% to 95.5% and from 10.5% to 90.5%, respectively). Visually, the sub-cluster tree topologies were hardly comparable between MLSA and MLEE, although only branches supported by BP above 70% were used. This inconsistency was confirmed by the SH test (p-value<0.05; Table S5) except for cluster II (*p*-value = 0.28). To assess whether the genetic structure within clusters was similar between MLSA and MLEE, the supertree approach was used to combine in a supertree all the MLSA and MLEE sub-trees corresponding to the main individual clusters. This method showed that the genetic structure within clusters was not consistent between the two classifications (CIC values lower than 40%). Only cluster II (*L. tropica*) exhibited a well conserved genetic structure between MLSA and MLEE as indicated by the many sub-groups that were conserved in the supertree (Figure S8; CIC value above 78%). The juxtaposition of the genetic data to the geographical data showed that the geographical origin of the genotypes was not randomly distributed (Figure 3). Although only the cluster II exhibited an evident correlation between the genetic structure and the geographical origin of the strains (see Discussion section). On the other hand, merging the MLEE-based groups into the MLSA-based cluster VII network showed clear inconsistencies (Figure 4). The sub-networks VIIb and VIIc were partially matched with the *L. donovani* and *L. infantum* taxa, respectively. However, the genotypes LST44, LST81 and LST140, which were identified by MLEE as *L. donovani*, were grouped within the VIIc sub-network and LST109 within the VIIb sub-network. The sub-network VIIa appeared to be a mixture of the three MLEE-based taxa *L. donovani*, *L. infantum* and *L. archibaldi*.

## Discussion

During the last century, an increasing number of species that belong to the *Leishmania* genus have been described, including about 20 species that are pathogenic for humans. These species are taxa that were defined using various epidemiological, clinical and mainly biochemical criteria and now their classification is debated either because it does not seem to be justified (*e.g.*, *L. killicki*, *L. archibaldi*) or because interspecific hybrids, which have been described even between distant taxa, could question their existence as species, at least according to the biological definition of species [4], [5].

In this study, the different probabilistic and topological approaches used allowed the definition of seven well individualized and well supported genetic clusters (I to VII). Although these clusters match quite well the currently used taxa (*i.e.*, species or species complexes, as discussed below), we decided to keep the numbering from I to VII throughout the analysis and discussion of our results, in order to avoid any *a priori* interference between our work and the classical *Leishmania* taxonomy.

### Interspecific hybridization in the *Leishmania* genus: no obvious trace in the analyzed genomes

One of the main results of this work is the remarkable congruence of the phylogenetic message of the analyzed loci. As shown in Figure 2, the structure of the networks obtained for loci 03.0980, 04.0580, 12.0010 and 31.0280 are almost identical. The structures of loci 10.0560, 14.0130 and 31.2610 are also quite similar to those of loci 03.0980, 04.0580, 12.0010 and 31.0280, although they are less resolved especially for clusters I and II. This lower resolution could be explained by a smaller number of informative sites for each of these loci (n = 41, 46 and 27, respectively; mean: 38) compared to loci 03.0980, 04.0580, 12.0010 and 31.0280 (n = 67, 71, 75 and 85, respectively; mean: 74). Nevertheless, the SH test did not show any contradiction between the phylogenetic signals of each of the seven loci (taken two by two) and the signals of each locus compared to the signal produced by the concatenated sequences (Figure 1).

This remarkable congruence was confirmed by both statistical (non-parametric SH test) and topological tests (PhySIC_IST). This result is surprising as hybrids between different *Leishmania* species have been described *in natura* [16]–[24]. These observations suggested that gene-flows could occur between species during evolution and that hybridization could be an important evolutionary mechanism for the adaptation of parasites to a variety of life cycles and conditions [50], [51]. The ensuing genome recombination should have given to each gene its own phylogenetic history. Conversely, our results seem to indicate that, although interspecific hybrids exist and can be stable over time, there is no obvious trace of genome recombination and allele introgression between different species. Due to the limited number of genes and strains analyzed in this study it is, however, not possible to exclude that genetic exchanges might act as an evolutionary driving force, particularly in restricted geographical area where true sympatric conditions may exist. It might well be that intra-specific genetic exchanges are much more frequent and much more difficult to detect than inter-specific exchanges.

The analysis of alleles shared by clusters I and II, which are sometimes intermingled (Figure S4B) or incompletely resolved (box-like structures in Figure 1) is in line with these conclusions. The genotype analysis in these two clusters identified 10 alleles that are shared by all strains and are specific to these two clusters, indicating a common phylogenetic origin of these alleles. However, surprisingly, 25 allelic positions segregated in cluster II but not in cluster I, and 10 positions segregated in cluster I but not in cluster II. Only positions 3491 and 3934 have alleles that segregate in both groups. Thus, although clusters I and II have a common phylogenetic origin and maybe a rather recent individualization, they do not seem to exchange alleles significantly. They might be in a divergent phase of evolution, although they can sometimes be found in sympatric association in Ethiopia and have a common vector *(Ph. Sergenti)* or reservoir *(Procavia capensis)* [52].

To our knowledge, comparable studies on other prokaryotic or eukaryotic organisms with proven existence of interspecific hybrids did not show such an important identity of the phylogenetic signal between *a priori* independent loci. To explain our results from a biological point of view, two hypotheses can be proposed: i) the elective loss of one of the two parents' entire genome in hybrid strains, or ii) the instability of hybrid genomes, which would only appear in exceptional conditions and would then be lost or non- adapted, unable to recombine or transmit new alleles through introgression in a variety of lineages. The experimental work carried out by Peacock et al. on the fate of *Trypanosoma brucei* hybrid cells during cycles in glossina and mice showing the elective loss of genetic material from one parent is in favor of the first hypothesis [53]. However these biases are not systematic, and considering the high number of interspecific hybrids described in *Leishmania*, this hypothesis seems little plausible. The second hypothesis is more interesting, but difficult to demonstrate. It supposes that a hybrid genome with a high nucleotide divergence between parental genomes might not persist in the long term. This could be due to disruptions in the expression of these allopolyploid genomes (transgressive gene regulation, alteration by cell size increase, non-additive expression, etc.), or even to anarchic mitotic segregation [54], [55].

### More evolution by mutation than recombination?

We did not detect any recombination signal whatever the approach used, except between the genotypes LST0045, LST0032 and LST0115. Conversely, we identified heterozygous positions in which only one of the two parental alleles was detected in the dataset (orphan allele), probably as a result of mutation rather than recombination events. Accumulation of independent mutations on each of the two homologous chromosomes has been observed in organisms with asexual reproduction, such as Bdelloids, and is usually described as the Meselson effect [56]. However this accumulation does not seem to be systematic and some intensive mechanisms of gene conversion could counteract this effect [57], [58]. Our results could also be explained by insufficient sampling and the existence of population subdivisions (Wahlund effect), particularly due to geographical or ecological isolation, as discussed below. The number of analyzed strains is probably not sufficient to investigate gene exchanges within *Leishmania* groups by classic population genetic approaches and the parental genotypes could have been lost in the natural population or misrepresented. This could explain the apparent contradiction between our MLSA-based results (mainly independent lineages that accumulate divergences without any sign of recent recombination) and the findings of many MLMT-based studies that often could define the structuration of various populations and showed exchanges or even fixed hybridization events [59]–[61]. In addition, some genotypes included in our dataset were identical, although the corresponding strains were collected over a long period of time. For example, the strains with the genotypes LST0001, LST0002, LST0010, LST0008, LST0005 and LST0007 were collected over 30, 29, 27, 26, 25 and 23 years respectively. This temporal stability is in agreement with results presented by Boité et al. on South American *Leishmania* strains belonging to the *Viannia* sub-genus and might reflect limited genetic recombination [13].

### Geographical distance and genetic distance: multiple patterns

The relationship between geographical distance and genetic distance is a complex issue for parasites hosted and transmitted by many mammalian and sandfly species. The parasite maintenance depends on its capacity to survive in a complex cycle that involves at least one long-lasting reservoir and a competent vector (adapted or permissive) [62]. Accordingly, the parasite distribution should be influenced mainly by the geographical distribution of reservoirs and vectors. However, the parasite capacity to jump from one cycle to another could largely modify this relationship and explain the unexpected spread of some strains.

The sample size of some clusters (III, IV and V, considered as *L. arabica*, *L. turanica* and *L. gerbilli*, respectively) was not big enough to detect reliably a geographical structuration. Seventeen of the 18 strains of cluster I (considered as *L. aethiopica*) come from a very restricted geographical area (Ethiopia) and are maintained in single cycles. They depend on reservoirs of the *Procaviidae* family, such as Rock Hyrax (*Procavia capensis*) and Bush Hyrax (*Heterohyrax brucei*), and are transmitted by vectors that are endemic in this area (*Ph. longipes* and *Ph. pedifer*) [63]. On the contrary, clusters II, VI and VII have a wide geographical distribution and their geographical structuration can be reliably analyzed.

Cluster II (considered to include *L. tropica* and *L. killicki*) is clearly genetically structured as previously reported [64]. This genetic sub-structuration is strongly correlated with the geographical origin for several groups of strains (cf. Figure 3). For instance, the related genotypes LST0068, LST0074 and LST0077 in Kenya or LST0059, LST0065 and LST0069 in Morocco have a strong genetic differentiation, suggesting that these groups of strains are diverging. An explanation would be that these parasites might depend on cycles involving wild animals living in very restricted biotopes, although humans have been considered as the reservoir of these parasites for a long time. Indeed, for genotypes LST0121 and LST0016, which correspond to Tunisian strains, the role of a small rodent (*Ctenodactylus gundi*) that lives in stone desert areas has recently been confirmed [65]. Similarly, *Procaviidae* are proven reservoirs of these parasites in Israel and East Africa (Kenya, Namibia) and other wild reservoirs remain to be identified [see 66]. However, this strong correlation between geography and genetic structure only concerns part of the strains included in cluster II. Our results also show that some strains that are genetically closely related are scattered in very wide geographical areas. Thus, the almost identical genotypes LST0034, LST0057, LST0058, LST0070, LST0085 and LST0115 correspond to strains from Morocco, Israel, Jordan, Greece and Turkey. As humans are the reservoir of these strains, and possibly dogs in Morocco, these strains could have been spread through movements of human populations and much faster than strains that depend on wild reservoirs, which are often very localized and isolated [67].

In cluster VI (considered as *L. major*), we observed a weak genetic structuration and a weak correlation with the geographical origin, except for Central Asian strains. This suggests that flows exist between the different foci. Indeed, identical genotypes are found in wide geographical areas. For instance, the genotype LST0002 comes from strains isolated in Senegal, Morocco, Algeria, Tunisia and Libya. This weak structuration is surprising because many rodent species that belong to at least nine different genera and with a variety of habitats and often a specific ecology have been described as reservoirs of parasites from this group [3]. However, some reservoirs with a wide distribution, such as the Libyan jird (*Meriones Libycus*) that is found from West Sahara to China, could facilitate the spread of parasites in different endemic areas [68]. Based on the analysis of coding sequences, Elfari and Al-Jawabreh defined three main populations corresponding to three geographical areas: Middle East, Africa and Central Asia (a little diversified group) [68], [69]. Similarly, we identified an African subgroup and a Near East/Middle East (Israel, Jordan, Egypt, Saudi Arabia) subgroup. Genotypes from Central Asia (LST0017, LST0018), which probably derived from Middle Eastern strains, are clearly diverging from cluster II. Accordingly, experimental studies (crossed infections) in which strains from Central Asia, Middle East or Africa were inoculated into reservoirs present in these different regions showed that *Rhombomys opimus* (greater gerbil), the main reservoir in Central Asia (present from the Caspian Sea to Mongolia), cannot be infected by strains from Africa or the Near/Middle East [68]. Our results also show that genotypes from Iran, Iraq and the Indian sub-continent are in an intermediary position between African and Near/Middle East groups. We do not have a convincing hypothesis to explain this distribution.

Cluster VII (*L. donovani*, *L. infantum* and *L. archibaldi*) seems to have a poor genetic structure, but can be roughly divided in two major groups: one gathering strains mainly from Eastern Africa (Kenya, Soudan, Ethiopia) and the other including strains from countries around the Mediterranean basin. Some strains from India and China are clearly genetically different (cf. Figure 3). Many population genetics studies (including comparisons of whole genomes) that focused on strains belonging to this cluster, usually at a smaller geographical scale, reported often contrasting/different description of the geographical structuration of populations and sub-populations [review in 70], [ 60], [ 61], [ 71], [ 72]. For instance, using similar MLMT approaches, Downing et al. showed that almost all of the 168 Indian strains under study were genetically identical (108 completely identical to each other). Conversely, Gelanew et al. found in 63 Ethiopian strains as many genotypes as the strains analyzed [11], [60]. Due to the insufficient number of polymorphisms (as compared to MLMT and entire genome sequencing) and the small number of samples from each country, our data cannot contribute to the debate at this smaller geographical scale. Conversely, at a larger geographical scale and notwithstanding possible sampling bias, our work shows two important points: i) highly divergent genotypes can be present in the same country, ii) the LST0001 and LST0003 genotypes are widespread (in 21 and 5 countries, respectively) and 56 of the 64 genotypes of cluster VII (are limited to a single country (Table S1). These results seem to indicate that the spread of strains in different countries is not even. This could not be directly explained by the important mobility of the two main reservoirs (humans in the Middle East and East Africa and dogs in the Mediterranean area) of cluster VII strains. It can be hypothesized that genotypes LST0001 and LST0003 might have specific features that allow them to efficiently adapt to various ecological conditions and to spread. Our results also show that, in a given place, the parasitic cycle is possible with parasites that are genetically very different, and that genetic homogeneity is not necessarily a consequence of the adaptation to a very specific cycle.

### Genetic data, biochemical characterization and taxonomy: a good correlation

Since 1990, the identification and classification of the *Leishmania* genus have mainly been based on the biochemical characterization of 15 isoenzyme systems (MLEE) and are generally well correlated with known epidemiological data on the different vectors and reservoirs [15]. However, several recent studies questioned the validity and practical interest of some *Leishmania* species [4], [5]. This controversy has important practical implications, particularly for the harmonization and standardization of therapeutic care of patients with leishmaniasis. The question of the criteria used to define *Leishmania* species is complicated both by the existence of interspecific hybrids and by the difficulty to show genetic exchanges within species.

This work was not carried out with the aim of proposing taxonomic changes. We chose i) to analyze *a priori* genetic data without considering the species names to avoid interferences between methods, and ii) to analyze the available isoenzymatic data for each strain to assess their correlation with the gathered genetic data. This correlation is on the whole very good. The analyzed Eurasian and African strains are related to 10 species according to the current biochemical classification and these 10 groups matched the seven genetic clusters defined by MLSA and are separated by large genetic distances (cf. Figure 1). Several molecular studies did not manage to clearly identify strains from cluster I/*L. aethiopica* and from cluster II/*L. tropica* [14], [73], [74]. Some of these results could be explained by an insufficient phylogenetic signal due to the small numbers of strains and/or markers. Our findings, and in particular the separated segregation of alleles between the seven clusters (cf. supra) would rather be in favor of a progressive genetic isolation between cluster I and II. Strains from cluster I/*L. aethiopica* might be distant descendants of ancestral populations that led to cluster II/*L.tropica* and that adapted to a very specific cycle (see the “Geographical distance and genetic distance” section above) and became confined to Ethiopia. From an ecological and epidemiological point of view, the localization of the vectors *Ph. longipes* and *Ph. Pedifer*, which are endemic in Ethiopian and Kenyan uplands (usually at altitudes above 1700 meters), could reflect this restricted distribution, although the tightness of such geographical barriers can sometimes be questioned as a unique explanatory mechanism [18], [52]. Similarly, the *L. killicki* species that was described by Rioux et al. based on biochemical and epidemiological criteria, is not clearly differentiated from other cluster II strains in our analysis [75]. However, although included in cluster II, strains that are described by biochemical criteria as *L. killicki* remained grouped in our analysis and could represent a branch located in Tunisia and Eastern Algeria and in the process of differentiating. The finding that their reservoir (*Ctenodactylus gundi*) is a small rodent living in mountainous and dry areas in these regions could partly explain the differentiation of this group [65]. However, this sub-cluster was genetically poorly differentiated and we would not consider *L. killicki* as a valid *Leishmania* species.

Clusters III/*L. arabica*, IV/*L. turanica* and V/*L. gerbilli* are clearly individualized and concordant, although they are represented by a small number of strains. Cluster VI and *L. major* match perfectly.

Clusters VII gathers strains belonging to three taxa (*L. donovani*, *L. infantum* and *L. archibaldi*). Their biochemical identification by electrophoretic mobility is based on a single polymorphism (275Tyr/275Asp) in the enzyme Glutamic oxaloacetic transaminase (GOT) (relative mobility: 113, 100 and 110, respectively; 110 appears to be the mobility of the heterozygous 100+113 alleles, F. Pratlong pers. comm.). All the strains analyzed in this study were characterized by this technique. Such a taxonomical classification based on a single amino acid polymorphism is controversial and could be prone to homoplasic mutations (see ref. 60 and Figure 4) [4], [5], [76]. In our analysis, cluster VII genotypes were roughly subdivided in three sub-networks. Sub-networks VIIa and VIIb almost exclusively included East African strains (Soudan, Ethiopia, Kenya), whereas sub-network VIIc included North African and Western European strains. Some genotypes from the Far East (China, India, Sri Lanka) were not included in these sub-clusters and are characterized by large genetic distances (*i.e.* LST0019, LST0037, LST00132 and LST0133 in Figure 4).

From a taxonomic point of view, sub-networks VIIb and VIIc partially match the taxa *L. donovani* and *L. infantum*, respectively. However, the genotypes LST0044, LST0081 and LST0140 that correspond to strains from Turkey, Ukraine and Yemen identified as *L. donovani* by biochemical methods were included in our analysis among strains identified as *L. infantum* (sub-network VIIc). This could be result of homoplasy at the *GOT* locus, as suggested by Jamjoon et al. to explain inconsistencies in the identification of East African strains [76]. The sub-network VIIa did not show any congruence with the biochemical classification. Strains identified as *L. donovani*, *L. infantum* or *L. archibaldi* are intermingled and are poorly differentiated from a genetic point of view. Piarroux et al. hypothesized that the taxon *L. archibaldi* might be a hybrid between *L. infantum* and *L. donovani*, based on the heterozygosity of the gene coding for the GOT enzyme [77]. Our results do not show a significantly high heterozygosity rate for the strains of this taxon. The validity of the taxon *L. archibaldi* was questioned by several genetic studies because it cannot be differentiated from *L. donovani* [review in 76]. Our results suggest that the biochemical characterization of strains included in this sub-network does not allow defining a taxon without ambiguity. This discordance could be due to homoplasy but also to the lack of robustness [49], [71], [76 and supra]. The *L. infantum* and *L. donovani* taxa, originally proposed to reflect different cycles and clinical manifestations, are in relative continuity and sometimes intermingled, in line with the term “*L. donovani* species complex” suggested by Lainson and Shaw 25 years ago [78], [79]. This might be due to ongoing exchanges between these taxa or to progressive differentiation, but without taxonomic and geographical discontinuity. This seems to contradict results of many studies using more variable markers, such as MLMT, and showing that an obvious structuration exists in this population, even sometimes at a very small scale [review in 61], [ 70]. Altogether, these data suggest that local barriers might be enough to maintain a detectable structuration; however, at a larger geographical and temporal scale, strains included in cluster VII might form a continuum.

### Conclusion

After more than 20 years of debate, the question of genetic exchanges between parasites of the *Leishmania* genus remains open. Although interspecific exchanges could be a possible source of plasticity and adaptation to new life conditions and they have been described *in natura*, they do not seem to have left detectable traces in the current genomes, at least in our dataset (222 *Leishmania* strains from Africa and Eurasia). Different *Leishmania* species might evolve through progressive divergence, by accumulating mutations and being structured through distinct parasitic cycles. Concomitantly, the dispersion of some genotypes in wide geographical areas also indicates a process of active diffusion, probably linked to the mobility of some reservoirs, such as humans or dogs, and to the parasite capacity to jump from a reservoir species to another.

From a taxonomic point of view, the strong genetic structuration of the different species does not question the current classification, except for species causing visceral forms of leishmaniasis (*L. donovani*, *L. infantum* and *L. archibaldi*) for which the current classification does not seem valid. Although these taxa cause different clinical forms and are maintained in different cycles, they are not clearly distinct and form a continuum, in line with the concept of “*L. donovani* species complex” already suggested for this group a long time ago. Our data also suggest that *L. killicki* should not be considered as a valid *Leishmania* species.

These results might have practical consequences regarding the molecular identification of *Leishmania* parasites and the subsequent therapeutic management.

Finally, our system provides an improved resolution in comparison to MLEE and could contribute to both a novel MLSA/MLST system and future *Leishmania* subgenus whole genome sequencing projects.

## Supporting Information

Figure S1 **Neighbor-Net networks of duplicated and non-duplicated sequences.** Neighbor-Net analysis of duplicated (A) and non-duplicated (B) concatenated nucleotide sequences based on uncorrected p-distance matrices.
(TIF)

Figure S2 **Split decomposition analysis.** Split decomposition analysis of the concatenated sequences. The numbering and color coding of the seven genetic clusters are similar to those in Figure 1. The bootstrap values (in percentage) supporting each cluster are indicated.
(TIF)

Figure S3 **Comparison of the ML and Bayesian trees.** The ML (A) and Bayesian (B) tree estimations of the concatenated nucleotides show similar topologies. The color coding is similar to the one in Figure 1. Bootstrap values (1000 replicates) and posterior probabilities are indicated on the ML and Bayesian tree topologies, respectively.
(TIF)

Figure S4 **Assessment of the topological congruence among the ML trees of the seven loci.** The ML tree for each locus (A) and the PhySIC_IST tree obtained by combining the ML tree topologies of the individual loci (B) are represented and color-coded according to Figure 1.
(TIF)

Figure S5 **Concatenated amino acid ML tree.** ML tree of the concatenated amino acid sequences. The color coding is as in Figure 1. Bootstrap values (1000 replicates) are indicated.
(TIF)

Figure S6 **Jumping of potential recombinant genotypes in cluster II.** Genotypes LST0032, LST0070 and LST0115 (indicated by solid stars) were grouped and supported by high BP on ML tree loci 03.0980, 14.0130, 31.0280 and 31.2610 and were reshuffled on loci 04.0580, 10.0560 and 12.0010. Genotypes of the seven clusters are color-coded according to Figure 1. BP (1,000 replicates) are shown at the nodes.
(TIF)

Figure S7 **Comparison of the MLSA and MLEE NJ trees.** The Neighbor-joining tree topologies of the concatenated nucleotide data (A) and isoenzymatic data (B) for 222 *Leishmania* strains are represented. The color coding is as in Figure 1. Bootstrap values (1000 replicates) are indicated.
(TIF)

Figure S8 **Topological comparison of the NJ trees for the MLSA cluster II and the MLEE** ***L. tropica*** **complex taxa.** NJ trees for the MLSA-based data (A) and the MLEE-based data (B) were built. The A and B topologies were combined in a PhySIC_IST supertree (C). LEM0617 was not inserted in the supertree, as its position was too uncertain (STC threshold 0.9). The color coding corresponds to the subgroups defined in C. Bootstrap values (1000 replicates) are indicated.
(TIF)

Table S1 **Information on the 222 strains used in this study.**
(PDF)

Table S2 **Statistical assessment of the congruence between the Maximum Likelihood (ML) and Bayesian tree topologies.** ^a^: Log Likelihood of the ML and Bayesian tree topologies based on the concatenated nucleotides sequences ^b^: differences in Log likelihood between the ML and Bayesian trees. The congruence between ML and Bayesian tree topologies was confirmed with the SH test (*p*-value = 0.65).
(PDF)

Table S3 **Statistical assessment of the congruence between the concatenated nucleotide and amino acid tree topologies.** ^a^: Log Likelihood of the ML tree topologies of the concatenated nucleotide sequences (concatenated nt) and the concatenated amino acid sequences (concatenated AA). ^b^: differences in Log likelihood between nucleotide and amino acid trees. The SH test indicated no significant differences in Log likelihood (p-value = 0.36).
(PDF)

Table S4 **Statistical assessment of the congruence between the MLSA and MLEE datasets.** ^a^: Log Likelihood of the MLSA and MLEE NJ tree topologies ^b^: differences in Log likelihood between MLSA and MLEE NJ trees. The overall congruence was assessed by comparison of the likelihood between the concatenated nucleotide (nt) Neighbor Joining (NJ) tree (MLSA) and the isoenzymatic data (MLEE). The SH test showed significant congruence between the MLSA and MLEE NJ trees (p-value = 0.16), indicating that the seven clusters were conserved with both approaches.
(PDF)

Table S5 **Statistical assessment of the congruence between individual MLSA clusters and MLEE groups.** ^a^: Log Likelihood of the individual NJ trees for the MLSA clusters and MLEE groups (only clusters I, II, VI and VII were analyzed). ^b^: pairwise differences in Log likelihood between individual NJ trees for the MLSA clusters and MLEE groups. Only cluster II showed significant congruence between the MLSA and MLEE approaches, as indicated by the SH test (p-value = 0.28).
(PDF)

## References

[1] Alvar J, Velez ID, Bern C, Herrero M, Desjeux P, et al (2012) Leishmaniasis worldwide and global estimates of its incidence. PLoS One 7: e35671. 22693548 10.1371/journal.pone.0035671 PMC3365071

[2] WHO (2010) Annul report. Geneva.

[3] Ashford RW (1996) Leishmaniasis reservoirs and their significance in control. Clin Dermatol 14: 523–532. 8889331 10.1016/0738-081x(96)00041-7

[4] Schonian G, Mauricio I, Cupolillo E (2010) Is it time to revise the nomenclature of *Leishmania*? Trends in Parasitology 26: 466–469. 20609626 10.1016/j.pt.2010.06.013

[5] Van der Auwera G, Fraga J, Montalvo AM, Dujardin JC (2011) *Leishmania* taxonomy up for promotion? Trends Parasitol 27: 49–50. 21147036 10.1016/j.pt.2010.11.007

[6] Akopyants NS, Kimblin N, Secundino N, Patrick R, Peters N, et al (2009) Demonstration of genetic exchange during cyclical development of *Leishmania* in the sand fly vector. Science 324: 265–268. 19359589 10.1126/science.1169464 PMC2729066

[7] Miles MA, Yeo M, Mauricio IL (2009) *Leishmania* exploit sex. Science 324: 187–189. 19359570 10.1126/science.1172789

[8] Ivens AC, Peacock CS, Worthey EA, Murphy L, Aggarwal G, et al (2005) The genome of the kinetoplastid parasite, *Leishmania major* . Science 309: 436–442. 16020728 10.1126/science.1112680 PMC1470643

[9] Peacock CS, Seeger K, Harris D, Murphy L, Ruiz JC, et al (2007) Comparative genomic analysis of three *Leishmania* species that cause diverse human disease. Nature Genetics 39: 839–847. 17572675 10.1038/ng2053 PMC2592530

[10] Rogers MB, Hilley JD, Dickens NJ, Wilkes J, Bates PA, et al (2011) Chromosome and gene copy number variation allow major structural change between species and strains of *Leishmania* . Genome Res 21: 2129–2142. 22038252 10.1101/gr.122945.111 PMC3227102

[11] Downing T, Stark O, Vanaerschot M, Imamura H, Sanders M, et al (2012) Genome-wide SNP and microsatellite variation illuminate population-level epidemiology in the *Leishmania* donovani species complex. Infect Genet Evol 12: 149–59. 22119748 10.1016/j.meegid.2011.11.005 PMC3315668

[12] Raymond F, Boisvert S, Roy G, Ritt JF, Legare D, et al (2012) Genome sequencing of the lizard parasite *Leishmania tarentolae* reveals loss of genes associated to the intracellular stage of human pathogenic species. Nucleic Acids Res 40 3: 1131–1147. 21998295 10.1093/nar/gkr834 PMC3273817

[13] Boité MC, Mauricio IL, Miles MA, Cupolillo E (2012) New insights on taxonomy, phylogeny and population genetics of *Leishmania* (*Viannia*) parasites based on multilocus sequence analysis. PLoS Negl Trop Dis 6 11: e1888. 23133690 10.1371/journal.pntd.0001888 PMC3486886

[14] Fraga J, Montalvo AM, De Doncker S, Dujardin JC, Van der Auwera G (2010) Phylogeny of *Leishmania* species based on the heat-shock protein 70 gene. Infect Genet Evol 10: 238–245. 19913110 10.1016/j.meegid.2009.11.007

[15] Rioux JA, Lanotte G, Serres E, Pratlong F, Bastien P, et al (1990) Taxonomy of *Leishmania*. Use of isoenzymes. Suggestions for a new classification. Ann Parasitol Hum Comp 65: 111–125. 2080829 10.1051/parasite/1990653111

[16] Kelly JM, Law JM, Chapman CJ, Van Eys GJ, Evans DA (1991) Evidence of genetic recombination in *Leishmania* . Mol Biochem Parasitol 46: 253–263. 1656255 10.1016/0166-6851(91)90049-c

[17] Ravel C, Cortes S, Pratlong F, Morio F, Dedet JP, et al (2006) First report of genetic hybrids between two very divergent *Leishmania* species: *Leishmania infantum* and *Leishmania major* . Int J Parasitol 36: 1383–1388. 16930606 10.1016/j.ijpara.2006.06.019

[18] Odiwuor S, De Doncker S, Maes I, Dujardin JC, Van der Auwera G (2011) Natural *Leishmania donovani*/*Leishmania aethiopica* hybrids identified from Ethiopia. Infect Genet Evol 11: 2113–2118. 21558020 10.1016/j.meegid.2011.04.026

[19] Dujardin JC, Banuls AL, Llanos-Cuentas A, Alvarez E, DeDoncker S, et al (1995) Putative *Leishmania* hybrids in the Eastern Andean valley of Huanuco, Peru. Acta Trop 59: 293–307. 8533665 10.1016/0001-706x(95)00094-u

[20] Nolder D, Roncal N, Davies CR, Llanos-Cuentas A, Miles MA (2007) Multiple hybrid genotypes of *Leishmania* (*Viannia*) in a focus of mucocutaneous Leishmaniasis. Am J Trop Med Hyg 76 3: 573–578. 17360886

[21] Cortes S, Esteves C, Maurício I, Maia C, Cristovão JM, et al (2012) In vitro and in vivo behaviour of sympatric *Leishmania* (*V.*) *braziliensis*, *L.* (*V.*) *peruviana* and their hybrids. Parasitology 139 2: 191–199. 22054424 10.1017/S0031182011001909

[22] Delgado O, Cupolillo E, Bonfante-Garrido R, Silva S, Belfort E, et al (1997) Cutaneous leishmaniasis in Venezuela caused by infection with a new hybrid between *Leishmania* (*Viannia*) *braziliensis* and *L.* (*V.*) *guyanensis* . Mem Inst Oswaldo Cruz 92: 581–582. 9566221 10.1590/s0074-02761997000500002

[23] Banuls AL, Guerrini F, Le Pont F, Barrera C, Espinel I, et al (1997) Evidence for hybridization by multilocus enzyme electrophoresis and random amplified polymorphic DNA between *Leishmania braziliensis* and *Leishmania panamensis*/*guyanensis* in Ecuador. J Eukaryot Microbiol 44: 408–411. 9304809 10.1111/j.1550-7408.1997.tb05716.x

[24] Belli AA, Miles MA, Kelly JM (1994) A putative *Leishmania panamensis*/*Leishmania braziliensis* hybrid is a causative agent of human cutaneous leishmaniasis in Nicaragua. Parasitology 109: 435–442. 7800411 10.1017/s0031182000080689

[25] Librado P, Rozas J (2009) DnaSP v5: a software for comprehensive analysis of DNA polymorphism data. Bioinformatics 25: 1451–1452. 19346325 10.1093/bioinformatics/btp187

[26] Tamura K, Dudley J, Nei M, Kumar S (2007) MEGA4: Molecular Evolutionary Genetics Analysis (MEGA) software version 4.0. Mol Biol Evol 24: 1596–1599. 17488738 10.1093/molbev/msm092

[27] Morrison DA (2010) Using data-display networks for exploratory data analysis in phylogenetic studies. Mol Biol Evol 27: 1044–1057. 20034996 10.1093/molbev/msp309

[28] Guindon S, Gascuel O (2003) A simple, fast and accurate method to estimate large phylogenies by maximum-likelihood. Systematic Biology 52: 696–704. 14530136 10.1080/10635150390235520

[29] Guindon S, Dufayard JF, Lefort V, Anisimova M, Hordijk W, et al (2010) New algorithms and methods to estimate maximum-likelihood phylogenies: assessing the performance of PhyML 3.0. Syst Biol 59 3: 307–321. 20525638 10.1093/sysbio/syq010

[30] Posada D (2008) jModelTest: phylogenetic model averaging. Mol Biol Evol 25: 1253–1256. 18397919 10.1093/molbev/msn083

[31] Nei M, Li WH (1979) Mathematical model for studying genetic variation in terms of restriction endonucleases. Proc Natl Acad Sci 76: 5269–5273. 291943 10.1073/pnas.76.10.5269 PMC413122

[32] Buntjer JB (2001) Phylogenetic Computer Tools (PhylTools). Version 1.32 for Windows. Wageningen: Wageningen University, Laboratory of Plant reeding.

[33] Legendre P, Legendre L (1998) Numerical Ecology, 2nd ed. Amsterdam: Elsevier Science.

[34] Felsenstein J (2004) PHYLIP (Phylogeny Inference Package) version 3.6 (computer program). Available: http://evolution.genetics.washington.edu/phylip.html.

[35] Huson DH, Bryant D (2006) Application of phylogenetic networks in evolutionary studies. Mol Biol Evol 23: 254–267. 16221896 10.1093/molbev/msj030

[36] Swofford DL (2002) PAUP*. Phylogenetic Aanalysis Using Parsimony (*and Other Methods). Version 4. Suderland, Massachusetts: Sinauer Associates.

[37] Scornavacca C, Berry V, Lefort V, Douzery EJP, Ranwez V (2008) PhySIC_IST: cleaning source trees to infer more informative supertrees. BMC Bioinformatics 9: 413. 18834542 10.1186/1471-2105-9-413 PMC2576265

[38] Le SQ, Gascuel O (2008) An improved general amino acid replacement matrix. Mol Biol Evol 25: 1307–1320. 18367465 10.1093/molbev/msn067

[39] Posada D, Crandall KA (2001) Evaluation of methods for detecting recombination from DNA sequences: Computer simulations. Proc Natl Acad Sci U S A 98: 13757–13762. 11717435 10.1073/pnas.241370698 PMC61114

[40] Bruen TC, Philippe H, Bryant D (2006) A simple and robust statistical test for detecting the presence of recombination. Genetics 172: 2665–2681. 16489234 10.1534/genetics.105.048975 PMC1456386

[41] Maynard Smith J (1992) Analyzing the mosaic structure of genes. J Mol Evol 34: 126–129. 1556748 10.1007/BF00182389

[42] Padidam M, Sawyer S, Fauquet CM (1999) Possible emergence of new geminiviruses by frequent recombination. Virology 265: 218–225. 10600594 10.1006/viro.1999.0056

[43] Boni MF, Posada D, Feldman MW (2007) An exact nonparametric method for inferring mosaic structure in sequence triplets. Genetics 176: 1035–1047. 17409078 10.1534/genetics.106.068874 PMC1894573

[44] Martin D, Rybicki E (2000) RDP: Detection of recombination amongst aligned sequences. Bioinformatics 16: 562–563. 10980155 10.1093/bioinformatics/16.6.562

[45] Martin DP, Lemey P, Lott M, Moulton V, Posada D, et al (2010) RDP3: a flexible and fast computer program for analyzing recombination. Bioinformatics 26: 2462–2463. 20798170 10.1093/bioinformatics/btq467 PMC2944210

[46] Lemey P, Salemi M, Vandamme AM (2009) The Phylogenetic Handbook: A Practical Approach to Phylogenetic Analysis and Hypothesis Testing. Cambridge: Cambridge University Press. pp. 519–548.

[47] Yeo M, Mauricio IL, Messenger LA, Lewis MD, Llewellyn MS, et al (2011) Multilocus sequence typing (MLST) for lineage assignment and high resolution diversity studies in *Trypanosoma cruzi* . PLoS Negl Trop Dis 5: e1049. 21713026 10.1371/journal.pntd.0001049 PMC3119646

[48] Zemanová E, Jirku M, Mauricio IL, Horák A, Miles M, et al (2007) The *Leishmania donovani* complex: genotypes of the five metabolic enzymes (ICD, ME, MPI, G6PDH, and FH), new targets for multilocus sequence typing. Int J Parasitol 37: 149–160. 17027989 10.1016/j.ijpara.2006.08.008

[49] Mauricio I, Yeo M, Baghaei M, Doto D, Pratlong F, et al (2006) Towards multilocus sequence typing in the *Leishmania donovani* complex: resolving genotypes and haplotypes for five polymorphic metabolic enzymes (ASAT, GPI, NH1, NH2, PGD). Int J Parasitol 36: 757–769. 16725143 10.1016/j.ijpara.2006.03.006

[50] Volf P, Benkova I, Myskova J, Sadlova J, Campino L, et al (2007) Increased transmission potential of *Leishmania major*/*Leishmania infantum* hybrids. Int J Parasitol 37: 589–593. 17376453 10.1016/j.ijpara.2007.02.002 PMC2839924

[51] Volf P, Sadlova J (2009) Sex in *Leishmania* . Science 324: 1644. 10.1126/science.324_1644b 19556486

[52] Gebre-Michael T, Balkew M, Ali A, Ludovisi A, Gramiccia M (2004) The isolation of *Leishmania tropica* and *L. aethiopica* from *Phlebotomus* (*Paraphlebotomus*) species (Diptera: Psychodidae) in the Awash Valley, northeastern Ethiopia. Trans R Soc Trop Med Hyg 98: 64–70. 14702839 10.1016/s0035-9203(03)00008-7

[53] Peacock L, Ferris V, Bailey M, Gibson W (2009) Intraclonal mating occurs during tsetse transmission of *Trypanosoma brucei* . Parasit Vectors 2: 43. 19772562 10.1186/1756-3305-2-43 PMC2758857

[54] Comai L (2005) The advantages and disadvantages of being polyploid. Nature Genet 6: 836–846. 10.1038/nrg1711 16304599

[55] Albertin W, Marullo M (2012) Polyploidy in fungi: evolution after whole-genome duplication. Proc Biol Sci 279: 2497–2509. 22492065 10.1098/rspb.2012.0434 PMC3350714

[56] Mark Welch D, Meselson M (2000) Evidence for the evolution of bdelloid rotifers without sexual reproduction or genetic exchange. Science 288: 1211–1215. 10817991 10.1126/science.288.5469.1211

[57] Omilian AR, Cristescu ME, Dudycha JL, Lynch M (2006) Ameiotic recombination in asexual lineages of Daphnia. Proc Natl Acad Sci USA 103: 18638–18643. 17121990 10.1073/pnas.0606435103 PMC1693715

[58] Rougeron V, De Meeûs T, Kako Ouraga S, Hide M, Bañuls AL (2010) “Everything you always wanted to know about sex (but were afraid to ask)” in *Leishmania* after two decades of laboratory and field analyses. PLoS Pathog 6: e1001004. 20808896 10.1371/journal.ppat.1001004 PMC2924324

[59] Chargui N, Amro A, Haouas N, Schonian G, Babba H, et al (2009) Population structure of Tunisian *Leishmania infantum* and evidence for the existence of hybrids and gene flow between genetically different populations. Int J Parasitol 39: 801–811. 19211023 10.1016/j.ijpara.2008.11.016

[60] Gelanew T, Kuhls K, Hurissa Z, Weldegebreal T, Hailu W, et al (2010) Inference of population structure of *Leishmania donovani* strains isolated from different Ethiopian visceral leishmaniasis endemic areas. PLoS Negl Trop Dis 4: e889. 21103373 10.1371/journal.pntd.0000889 PMC2982834

[61] Rougeron V, De Meeûs T, Hide M, Le Falher G, Bucheton B, et al (2011) Multifaceted Population Structure and Reproductive Strategy in *Leishmania donovani* Complex in One Sudanese Village. PLoS Negl Trop Dis 5 12: e1448. 22206035 10.1371/journal.pntd.0001448 PMC3243727

[62] Myskova J, Svobodova M, Beverley SM, Volf P (2007) A lipophosphoglycan-independent development of *Leishmania* in permissive sand flies. Microbes Infect 9: 317–324. 17307009 10.1016/j.micinf.2006.12.010 PMC2839925

[63] Ashford RW, Bray MA, Hutchinson MP, Bray RS (1973) The epidemiology of cutaneous leishmaniasis in Ethiopia. Trans R Soc Trop Med Hyg 67: 568–601. 4150462 10.1016/0035-9203(73)90088-6

[64] Schwenkenbecher JM, Wirth T, Schnur LF, Jaffe CL, Schallig H, et al (2006) Microsatellite analysis reveals genetic structure of *Leishmania tropica* . Int J Parasitol 36: 237–246. 16307745 10.1016/j.ijpara.2005.09.010

[65] Jaouadi K, Haouas N, Chaara D, Gorcii M, Chargui N, et al (2011) First detection of *Leishmania killicki* (Kinetoplastida, Trypanosomatidae) in *Ctenodactylus gundi* (Rodentia, Ctenodactylidae), a possible reservoir of human cutaneous leishmaniasis in Tunisia. Parasit Vectors 4: 159. 21834996 10.1186/1756-3305-4-159 PMC3162927

[66] Talmi-Frank D, Kedem-Vaanunu N, King R, Bar-Gal GK, Edery N, et al (2010) *Leishmania tropica* infection in golden jackals and red foxes. Emerg Infect Dis 16: 1973–1975. 21122235 10.3201/eid1612.100953 PMC3294571

[67] Dereure J, Rioux JA, Gallego M, Perieres J, Pratlong F, et al (1991) *Leishmania tropica* in Morocco: infection in dogs. Trans R Soc Trop Med Hyg 85: 595. 1780983 10.1016/0035-9203(91)90356-4

[68] Elfari M, Schnur LF, Strelkova MV, Eisenberger CL, Jacobson RL, et al (2005) Genetic and biological diversity among populations of *Leishmania major* from Central Asia, the Middle East and Africa. Microbes Infect 7: 93–103. 15716069 10.1016/j.micinf.2004.09.010

[69] Al-Jawabreh A, Diezmann S, Muller M, Wirth T, Schnur LF, et al (2008) Identification of geographically distributed sub-populations of *Leishmania* (*Leishmania*) *major* by microsatellite analysis. BMC Evol Biol 8: 183. 18577226 10.1186/1471-2148-8-183 PMC2447845

[70] Schönian G, Kuhls K, Mauricio IL (2011) Molecular approaches for a better understanding of the epidemiology and population genetics of *Leishmania* . Parasitology 138: 405–425. 21078222 10.1017/S0031182010001538

[71] Alam MZ, Kuhls K, Schweynoch C, Sundar S, Rijal S, et al (2009) Multilocus microsatellite typing (MLMT) reveals genetic homogeneity of *Leishmania* donovani strains in the Indian subcontinent. Infect Genet Evol 9: 24–31. 18957333 10.1016/j.meegid.2008.09.005

[72] Downing T, Imamura H, Decuypere S, Clark TG, Coombs GH, et al (2011) Whole genome sequencing of multiple *Leishmania donovani* clinical isolates provides insights into population structure and mechanisms of drug resistance. Genome Res 21 12: 2143–2156. 22038251 10.1101/gr.123430.111 PMC3227103

[73] Mauricio IL, Gaunt MW, Stothard JR, Miles MA (2007) Glycoprotein 63 (gp63) genes show gene conversion and reveal the evolution of Old World *Leishmania* . Int J Parasitol 37: 565–576. 17280675 10.1016/j.ijpara.2006.11.020

[74] Asato Y, Oshiro M, Myint CK, Yamamoto Y, Kato H, et al (2009) Phylogenic analysis of the genus *Leishmania* by cytochrome b gene sequencing. Exp Parasitol 121: 352–361. 19159626 10.1016/j.exppara.2008.12.013

[75] Rioux JA, Lanotte G, Pratlong F (1986) *Leishmania killicki n.sp.* (Kinetoplastida-Trypanosomatidae). In: *Leishmania*, taxonomie et phylogenese. Applications eco-epidemiologiques (ed. JA Rioux) IMEEE, Montpellier, France, 139–142.

[76] Jamjoom MB, Ashford RW, Bates PA, Chance ML, Kemp SJ, et al (2004) *Leishmania donovani* is the only cause of visceral leishmaniasis in East Africa; previous descriptions of *L. infantum* and “*L. archibaldi*” from this region are a consequence of convergent evolution in the isoenzyme data. Parasitology 129: 399–409. 15521628 10.1017/s0031182004005955

[77] Piarroux R, Trouvé V, Pratlong F, Martini A, Lambert M, Rioux JA (1994) The use of isoelectric focusing on polyacrylamide gel for the enzymatic analysis of ‘Old World’ *Leishmania* species. Trans R Soc Trop Med Hyg 88: 475–478. 7570849 10.1016/0035-9203(94)90439-1

[78] Lainson R, Shaw JJ (1987) Evolution classification and geographical distribution. In: The Leishmaniases in Biology and Medicine (W Peters and R. Killick Kendrick Ed.) Academic Press Inc., London.

[79] Almeida LA, Araujo R (2013) Highlights on molecular identification of closely related species. Infect Genet Evol 13: 67–75. 22982158 10.1016/j.meegid.2012.08.011

