# Supplementary figures and images for: Genetic Structure and Evolution of the Leishmania Genus in Africa and Eurasia: What Does MLSA Tell Us
Source: PLoS Negl Trop Dis. 2013 Jun 13;7(6):e2255. doi: 10.1371/journal.pntd.0002255 (PMC3681676; doi:10.1371/journal.pntd.0002255)

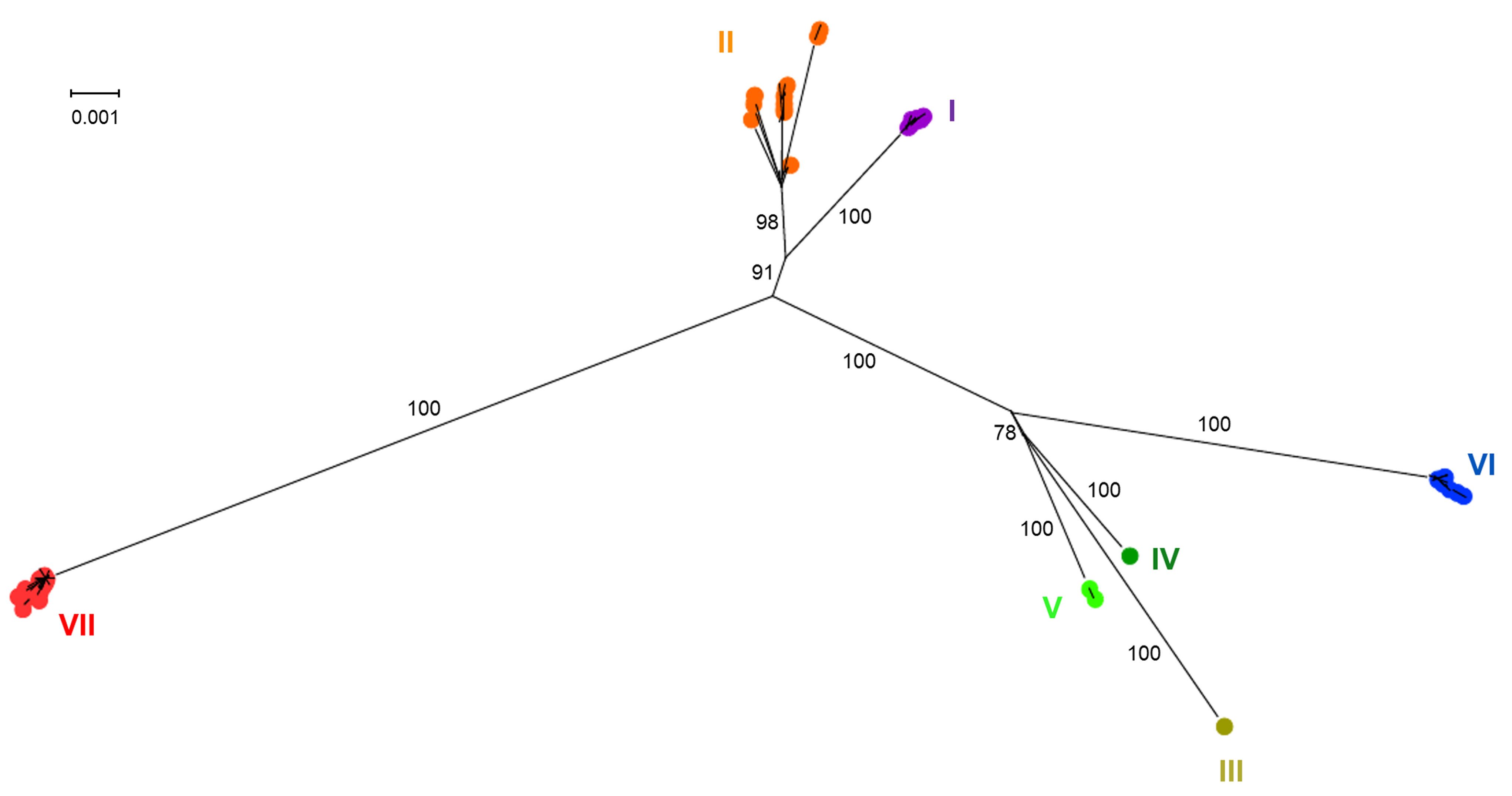

Supplement: Figure S1 — Neighbor-Net networks of duplicated and non-duplicated sequences. Neighbor-Net analysis of duplicated (A) and non-duplicated (B) concatenated nucleotide sequences based on uncorrected p-distance matrices. (TIF) [file pntd.0002255.s001.tif]

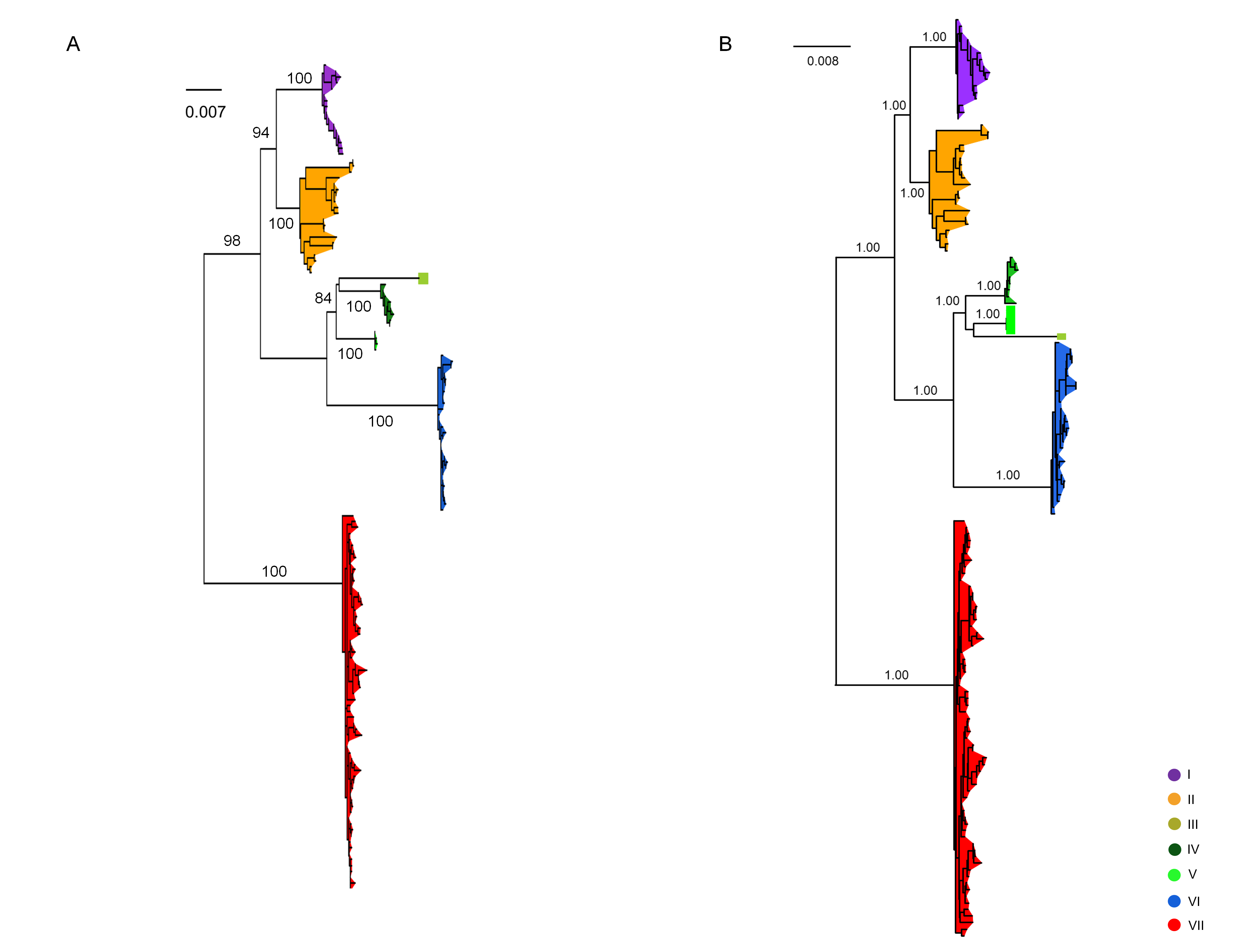

Supplement: Figure S2 — Split decomposition analysis. Split decomposition analysis of the concatenated sequences. The numbering and color coding of the seven genetic clusters are similar to those in Figure 1. The bootstrap values (in percentage) supporting each cluster are indicated. (TIF) [file pntd.0002255.s002.tif]

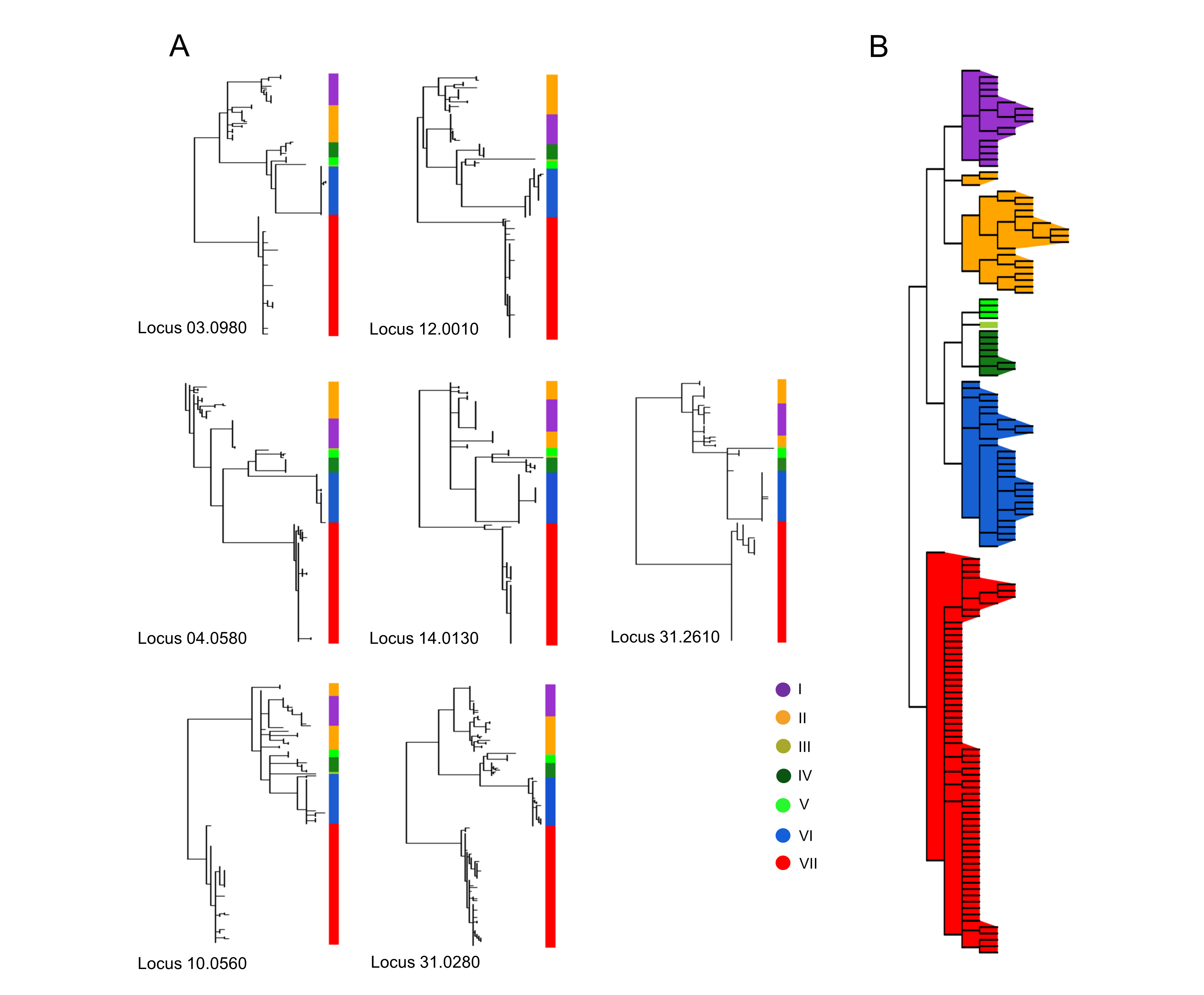

Supplement: Figure S3 — Comparison of the ML and Bayesian trees. The ML (A) and Bayesian (B) tree estimations of the concatenated nucleotides show similar topologies. The color coding is similar to the one in Figure 1. Bootstrap values (1000 replicates) and posterior probabilities are indicated on the ML and Bayesian tree topologies, respectively. (TIF) [file pntd.0002255.s003.tif]

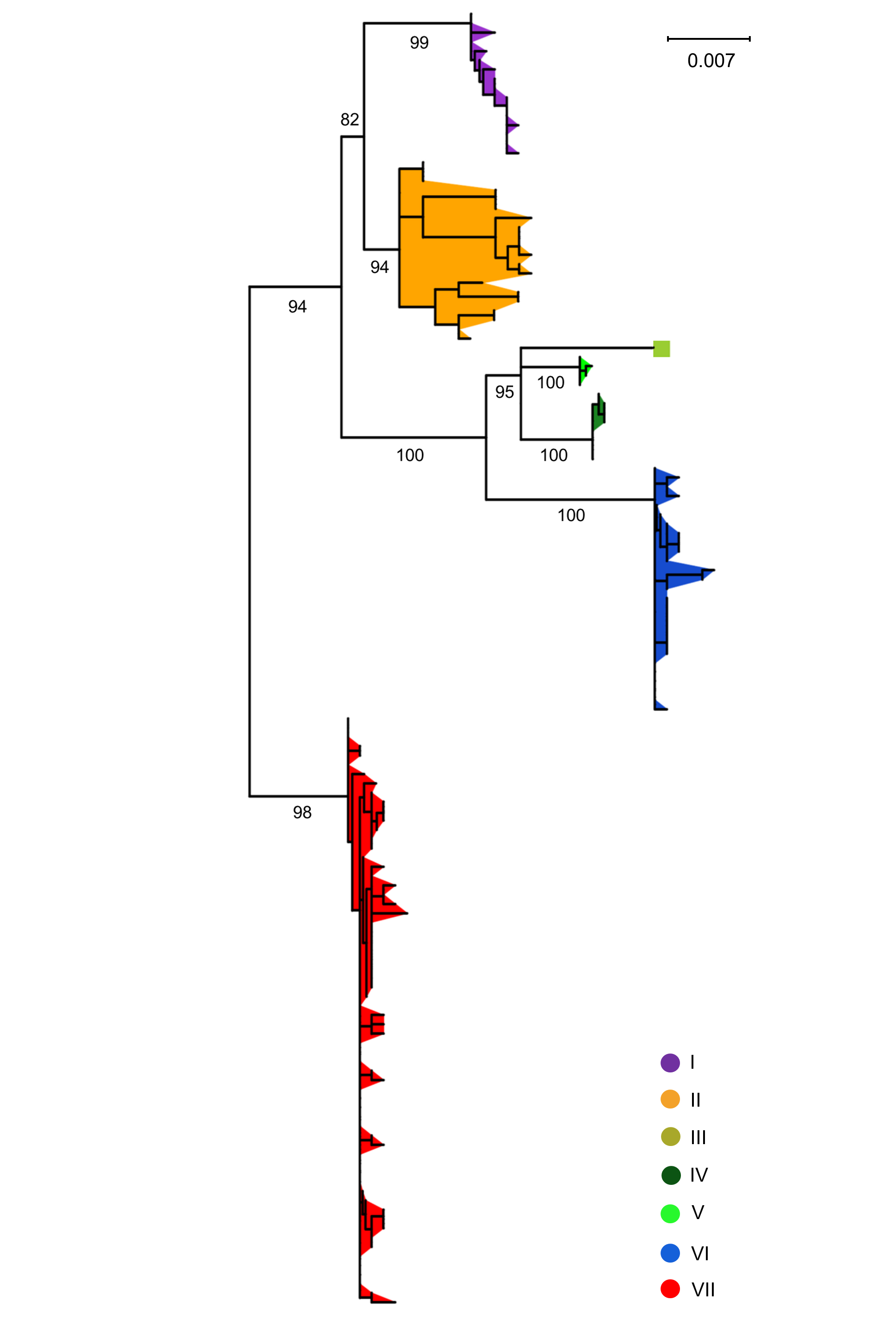

Supplement: Figure S4 — Assessment of the topological congruence among the ML trees of the seven loci. The ML tree for each locus (A) and the PhySIC_IST tree obtained by combining the ML tree topologies of the individual loci (B) are represented and color-coded according to Figure 1. (TIF) [file pntd.0002255.s004.tif]

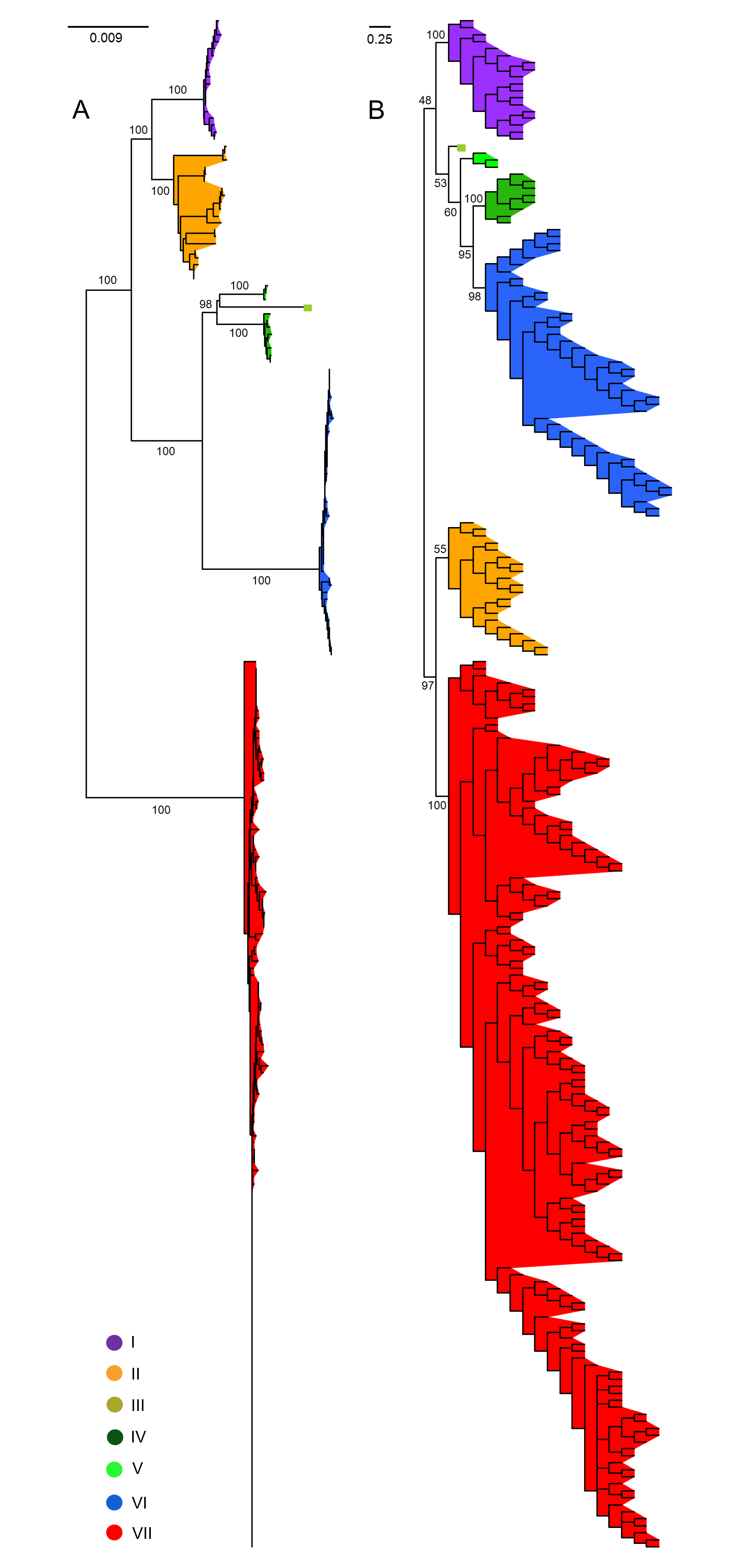

Supplement: Figure S5 — Concatenated amino acid ML tree. ML tree of the concatenated amino acid sequences. The color coding is as in Figure 1. Bootstrap values (1000 replicates) are indicated. (TIF) [file pntd.0002255.s005.tif]

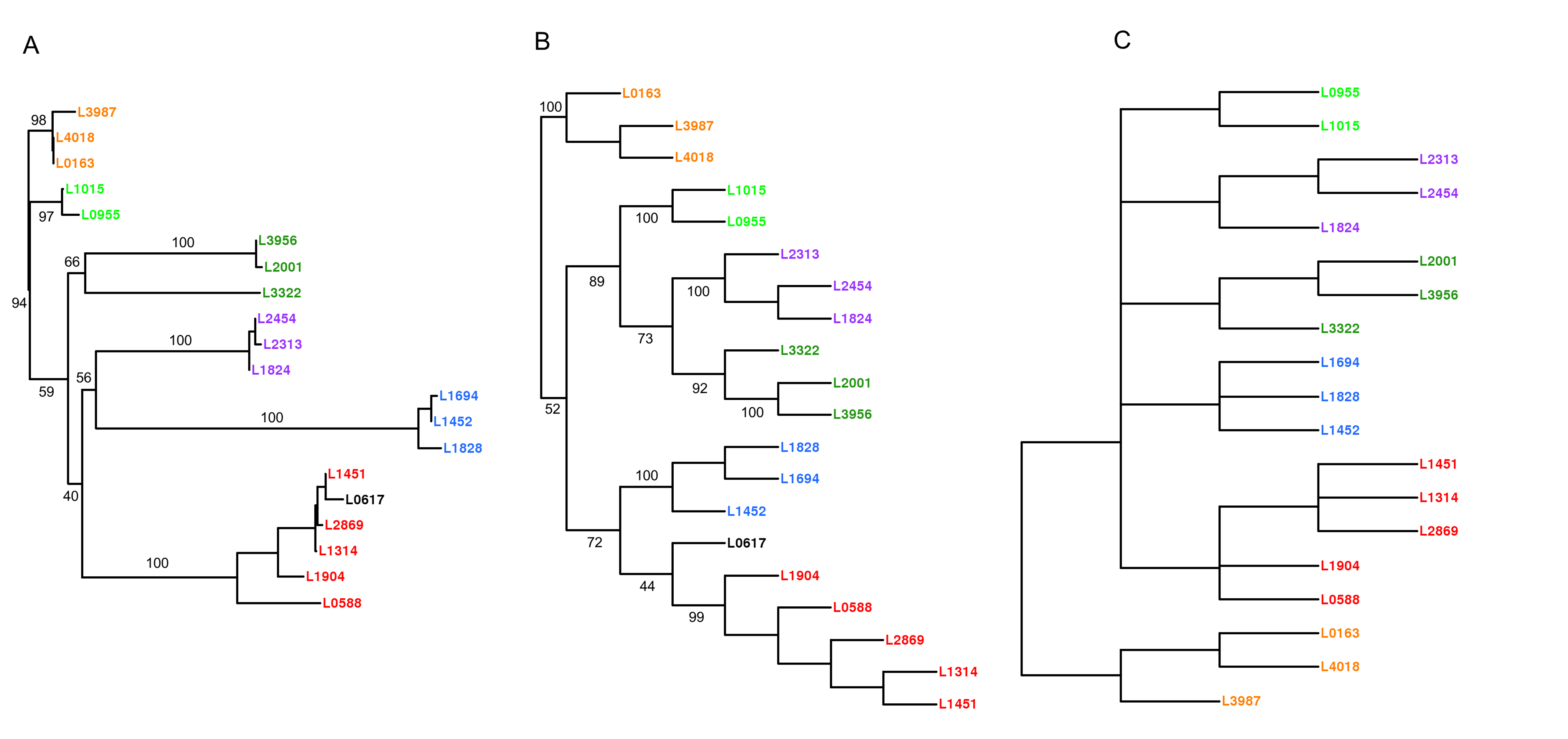

Supplement: Figure S6 — Jumping of potential recombinant genotypes in cluster II. Genotypes LST0032, LST0070 and LST0115 (indicated by solid stars) were grouped and supported by high BP on ML tree loci 03.0980, 14.0130, 31.0280 and 31.2610 and were reshuffled on loci 04.0580, 10.0560 and 12.0010. Genotypes of the seven clusters are color-coded according to Figure 1. BP (1,000 replicates) are shown at the nodes. (TIF) [file pntd.0002255.s006.tif]

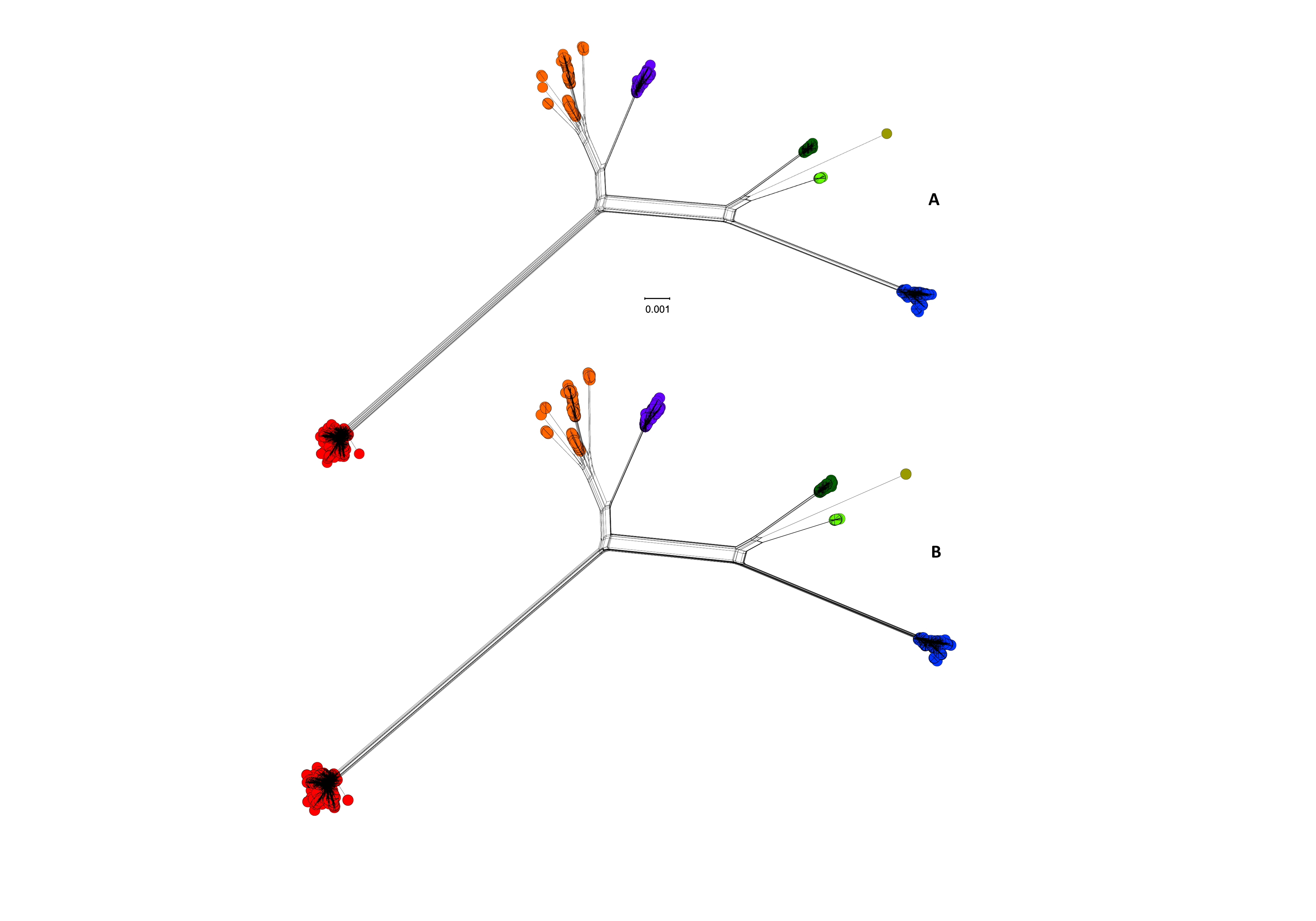

Supplement: Figure S7 — Comparison of the MLSA and MLEE NJ trees. The Neighbor-joining tree topologies of the concatenated nucleotide data (A) and isoenzymatic data (B) for 222 Leishmania strains are represented. The color coding is as in Figure 1. Bootstrap values (1000 replicates) are indicated. (TIF) [file pntd.0002255.s007.tif]

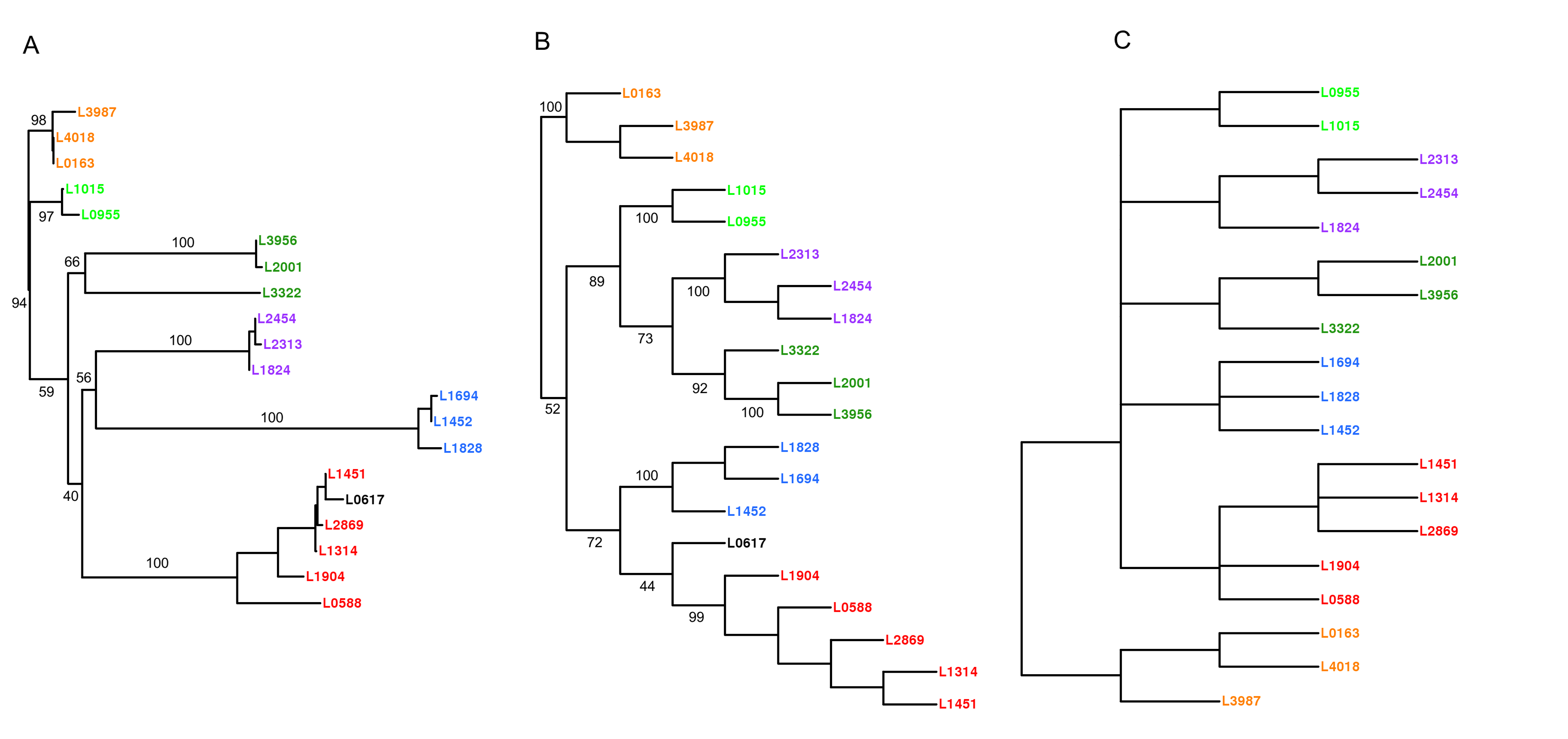

Supplement: Figure S8 — Topological comparison of the NJ trees for the MLSA cluster II and the MLEE L. tropica complex taxa. NJ trees for the MLSA-based data (A) and the MLEE-based data (B) were built. The A and B topologies were combined in a PhySIC_IST supertree (C). LEM0617 was not inserted in the supertree, as its position was too uncertain (STC threshold 0.9). The color coding corresponds to the subgroups defined in C. Bootstrap values (1000 replicates) are indicated. (TIF) [file pntd.0002255.s008.tif]
