# Supplementary material for: Genetic Structure and Evolution of the Leishmania Genus in Africa and Eurasia: What Does MLSA Tell Us
Source: PLoS Negl Trop Dis. 2013 Jun 13;7(6):e2255. doi: 10.1371/journal.pntd.0002255 (PMC3681676; doi:10.1371/journal.pntd.0002255)
Supplement: Table S1 — Information on the 222 strains used in this study. (PDF) [file pntd.0002255.s009.pdf]

Table S1. Information on the 222 strains used in this study.

LEM	WHO code	MLEE-based taxa	Country	Subregion	Zymodeme	Host	MLSA-based clusters	Genotype
**L0144**	MHOM/ET/72/L100	*L. aethiopica*	Ethiopia	E. AF	MON-14	*Homo sapiens*	Grp_I	LST0009
**L3348**	MHOM/ET/81/921-81	*L. aethiopica*	Ethiopia	E. AF	MON-261	*Homo sapiens*	Grp_I	LST0009
**L3353**	MHOM/ET/82/652-82a	*L. aethiopica*	Ethiopia	E. AF	MON-243	*Homo sapiens*	Grp_I	LST0009
**L0145**	MHOM/KE/71/KPS-H2	*L. aethiopica*	Kenya	E. AF	MON-14	*Homo sapiens*	Grp_I	LST0020
**L0615**	MHOM/ET/82/101-82	*L. aethiopica*	Ethiopia	E. AF	MON-69	*Homo sapiens*	Grp_I	LST0033
**L0618**	MHOM/ET/83/68-83	*L. aethiopica*	Ethiopia	E. AF	MON-70	*Homo sapiens*	Grp_I	LST0035
**L1067**	MHOM/ET/63/L28	*L. aethiopica*	Ethiopia	E. AF	MON-250	*Homo sapiens*	Grp_I	LST0051
**L1118**	MHOM/ET/83/130-83	*L. aethiopica*	Ethiopia	E. AF	MON-239	*Homo sapiens*	Grp_I	LST0053
**L1119**	MHOM/ET/83/103-83	*L. aethiopica*	Ethiopia	E. AF	MON-249	*Homo sapiens*	Grp_I	LST0054
**L1517**	MHOM/ET/89/LEM1517	*L. aethiopica*	Ethiopia	E. AF	MON-241	*Homo sapiens*	Grp_I	LST0060
**L1636**	MHOM/ET/83/85-83	*L. aethiopica*	Ethiopia	E. AF	MON-242	*Homo sapiens*	Grp_I	LST0063
**L1647**	MHOM/ET/72/L127	*L. aethiopica*	Ethiopia	E. AF	MON-247	*Homo sapiens*	Grp_I	LST0064
**L2357**	MHOM/ET/90/DISKO	*L. aethiopica*	Ethiopia	E. AF	MON-251	*Homo sapiens*	Grp_I	LST0075
**L2358**	MHOM/ET/91/KASSAYE	*L. aethiopica*	Ethiopia	E. AF	MON-248	*Homo sapiens*	Grp_I	LST0076
**L3351**	MHOM/ET/70/L96	*L. aethiopica*	Ethiopia	E. AF	MON-260	*Homo sapiens*	Grp_I	LST0095
**L3362**	MHOM/ET/82/741-82	*L. aethiopica*	Ethiopia	E. AF	MON-240	*Homo sapiens*	Grp_I	LST0096
**L3363**	MHOM/ET/81/1091-81	*L. aethiopica*	Ethiopia	E. AF	MON-245	*Homo sapiens*	Grp_I	LST0097
**L3364**	MPRV/ET/71/L111	*L. aethiopica*	Ethiopia	E. AF	MON-262	*Procavia habessinica*	Grp_I	LST0098
**L0163**	MHOM/TN/80/LEM163	*L. killicki*	Tunisia	N. AF	MON-8	*Homo sapiens*	Grp_II	LST0016
**L4018**	MHOM/TN/2000/26LC	*L. killicki*	Tunisia	N. AF	MON-8	*Homo sapiens*	Grp_II	LST0016
**L0588**	MHOM/GR/82/SER-L60	*L. tropica*	Greece	S.E	MON-57	*Homo sapiens*	Grp_II	LST0032
**L0617**	MHOM/IL/80/SINGER	*L. tropica*	Israel	M.E	MON-54	*Homo sapiens*	Grp_II	LST0034
**L0955**	MHOM/YE/86/LEM955	*L. tropica*	Yemen	M.E	MON-71	*Homo sapiens*	Grp_II	LST0045
**L1015**	MHOM/YE/86/LEM1015	*L. tropica*	Yemen	M.E	MON-71	*Homo sapiens*	Grp_II	LST0048
**L1314**	MHOM/MA/88/LEM1314	*L. tropica*	Morocco	N. AF	MON-102	*Homo sapiens*	Grp_II	LST0057
**L1451**	MHOM/MA/88/LEM1451	*L. tropica*	Morocco	N. AF	MON-112	*Homo sapiens*	Grp_II	LST0058
**L1452**	MHOM/MA/88/LEM1452	*L. tropica*	Morocco	N. AF	MON-107	*Homo sapiens*	Grp_II	LST0059
**L1694**	ISER/MA/89/LEM1694	*L. tropica*	Morocco	N. AF	MON-123	*Phlebotomus sergenti*	Grp_II	LST0065
**L1824**	MHOM/KE/86/EB103	*L. tropica*	Kenya	E. AF	MON-119	*Homo sapiens*	Grp_II	LST0068
**L1828**	ISER/MA/89/LEM1828	*L. tropica*	Morocco	N. AF	MON-122	*Phlebotomus sergenti*	Grp_II	LST0069
**L1904**	MHOM/GR/88/LA615	*L. tropica*	Greece	S.E	MON-114	*Homo sapiens*	Grp_II	LST0070
**L2001**	MHOM/EG/90/LPN65	*L. tropica*	Egypt	M.E	MON-137	*Homo sapiens*	Grp_II	LST0071
**L2313**	IGUG/KE/91/000	*L. tropica*	Kenya	E. AF	MON-119	*Homo sapiens*	Grp_II	LST0074

**L2454**	MHOM/KE/92/EB000	*L. tropica*	Kenya	E. AF	MON-119	*Homo sapiens*	Grp_II	LST0077
**L2869**	MHOM/JO/93/JH67	*L. tropica*	Jordan	M.E	MON-200	*Homo sapiens*	Grp_II	LST0085
**L3322**	MHOM/JO/96/JH-88	*L. tropica*	Jordan	M.E	MON-265	*Homo sapiens*	Grp_II	LST0094
**L3919**	MHOM/TR/99/LSL43	*L. tropica*	Turkey	M.E	MON-ND	*Homo sapiens*	Grp_II	LST0115
**L3956**	MHOM/IL/96/LRC-L691	*L. tropica*	Israel	M.E	MON-137	*Homo sapiens*	Grp_II	LST0118
**L3987**	MHOM/TN/2000/000a	*L. killicki*	Tunisia	N. AF	MON-8	*Homo sapiens*	Grp_II	LST0121
**L1108**	MPSA/SA/83/JISH220	*L. arabica*	Saudi Arabia	M.E	MON-99	*Psammomys*	Grp_III	LST0052
**L0423**	MRHO/SU/65/VL	*L. turanica*	Turkmenistan	C.A	MON-21	*Rhombomys opimus*	Grp_IV	LST0025
**L0558**	MRHO/SU/74/95A	*L. turanica*	Turkmenistan	C.A	MON-64	*Rhombomys opimus*	Grp_IV	LST0030
**L0563**	MMEL/SU/79/MEL	*L. turanica*	Georgia	W.A	MON-65	*Meles meles*	Grp_IV	LST0031
**L3408**	MRHO/SU/87/KD-87527L	*L. turanica*	Uzbekistan	C.A	MON-271	*Rhombomys opimus*	Grp_IV	LST0101
**L3411**	MRHO/SU/86/KD-86235	*L. turanica*	Uzbekistan	C.A	MON-270	*Rhombomys opimus*	Grp_IV	LST0103
**L3414**	MRHO/SU/95/T-9551R	*L. turanica*	Turkmenistan	C.A	MON-233	*Rhombomys opimus*	Grp_IV	LST0104
**L3415**	MRHO/MN/86/MNR-14	*L. turanica*	Mongolia	E.A	MON-234	*Rhombomys opimus*	Grp_IV	LST0105
**L3418**	MRHO/MN/83/MNR-6	*L. turanica*	Mongolia	E.A	MON-65	*Rhombomys opimus*	Grp_IV	LST0106
**L0452**	MRHO/CN/60/GERBILLI	*L. gerbilli*	China	E.A	MON-22	*Rhombomys opimus*	Grp_V	LST0027
**L3390**	MRHO/SU/87/E-11	*L. gerbilli*	Kazakhstan	C.A	MON-272	*Rhombomys opimus*	Grp_V	LST0099
**L3391**	MRHO/SU/95/T-9520	*L. gerbilli*	Turkmenistan	C.A	MON-ND	*Rhombomys opimus*	Grp_V	LST0100
**L3410**	MRHO/SU/87/KD-87542R	*L. gerbilli*	Uzbekistan	C.A	MON-273	*Rhombomys opimus*	Grp_V	LST0102
**L0133**	MHOM/LY/75/ASHFORD55	*L. major*	Libya	N. AF	MON-25	*Homo sapiens*	Grp_VI	LST0002
**L0309**	MMER/MA/81/LEM309	*L. major*	Morocco	N. AF	MON-25	*Meriones shawi*	Grp_VI	LST0002
**L1616**	MHOM/SN/89/CRE9	*L. major*	Senegal	W.AF	MON-25	*Homo sapiens*	Grp_VI	LST0002
**L1637**	MHOM/DZ/89/LIPA228	*L. major*	Algeria	N. AF	MON-25	*Homo sapiens*	Grp_VI	LST0002
**L3536**	MHOM/DZ/98/DPPE26	*L. major*	Algeria	N. AF	MON-269	*Homo sapiens*	Grp_VI	LST0002
**L4746**	MHOM/TN/2004/LEI21	*L. major*	Tunisia	N. AF	MON-25	*Homo sapiens*	Grp_VI	LST0002
**L0155**	MHOM/ML/80/LEM155	*L. major*	Mali	W.AF	MON-25	*Homo sapiens*	Grp_VI	LST0005
**L2389**	IDUB/ET/90/TESHOME202	*L. major*	Ethiopia	E. AF	MON-74	*Phlebotomus duboscqi*	Grp_VI	LST0005
**L2574**	MHOM/PT/92/CRE26	*L. major*	Portugal	S.E	MON-74	*Homo sapiens*	Grp_VI	LST0005
**L4812**	MHOM/00/2004/LEI26	*L. major*	Burkina Faso	W.AF	MON-26	*Homo sapiens*	Grp_VI	LST0005
**L4935**	MHOM/MR/2005/LPC8	*L. major*	Mauritania	W.AF	MON-26	*Homo sapiens*	Grp_VI	LST0005
**L3522**	MHOM/SN/98/LMM1	*L. major*	Senegal	W.AF	MON-117	*Homo sapiens*	Grp_VI	LST0006
**L4308**	MHOM/MR/2001/CRE118	*L. major*	Mauritania	W.AF	MON-74	*Homo sapiens*	Grp_VI	LST0006
**L4495**	MHOM/ML/2002/CRE125	*L. major*	Mali	W.AF	MON-74	*Homo sapiens*	Grp_VI	LST0006
**L4708**	MHOM/DZ/2003/CRE138-ER	*L. major*	Algeria	N. AF	MON-74	*Homo sapiens*	Grp_VI	LST0006
**L0755**	MHOM/IL/67/JERICHOII	*L. major*	Israel	M.E	MON-26	*Homo sapiens*	Grp_VI	LST0008
**L2699**	MHOM/SA/93/DPPE19	*L. major*	Saudi Arabia	M.E	MON-26	*Homo sapiens*	Grp_VI	LST0008
**L0062**	MHOM/YE/76/LEM62	*L. major*	Yemen	M.E	MON-26	*Homo sapiens*	Grp_VI	LST0011
**L0466**	MHOM/IL/83/IL24	*L. major*	Israel	M.E	MON-66	*Homo sapiens*	Grp_VI	LST0011

**L1159**	MTAT/KE/00/T4	*L. major*	Kenya	E. AF	MON-74	*Tatera sp.*	Grp_VI	LST0012
**L1776**	MHOM/MR/89/LIPA225	*L. major*	Mauritania	W.AF	MON-117	*Homo sapiens*	Grp_VI	LST0012
**L0129**	MRHO/SU/59/P-STRAIN	*L. major*	Uzbekistan	C.A	MON-4	*Rhombomys opimus*	Grp_VI	LST0017
**L0131**	MHOM/SU/73/29-ASKH	*L. major*	Turkmenistan	C.A	MON-26	*Homo sapiens*	Grp_VI	LST0018
**L0146**	MMER/IN/73/GTBM	*L. major*	India	I.S	MON-23	*Meriones hurrianae*	Grp_VI	LST0021
**L0328**	MHOM/EG/82/ALY	*L. major*	Egypt	M.E	MON-26	*Homo sapiens*	Grp_VI	LST0023
**L0468**	MHOM/IL/83/IL32	*L. major*	Israel	M.E	MON-68	*Homo sapiens*	Grp_VI	LST0028
**L0766**	MCAN/EG/83/ASC-D1	*L. major*	Egypt	M.E	MON-74	*Canis familiaris*	Grp_VI	LST0041
**L0858**	MHOM/IQ/86/CRE1	*L. major*	Iraq	M.E	MON-26	*Homo sapiens*	Grp_VI	LST0042
**L1558**	MHOM/IL/89/CRE7	*L. major*	Israel	M.E	MON-103	*Homo sapiens*	Grp_VI	LST0061
**L2892**	MHOM/TD/92/ISS728	*L. major*	Chad	C.AF	MON-196	*Homo sapiens*	Grp_VI	LST0087
**L3102**	MHOM/JO/90/JH39	*L. major*	Jordan	M.E	MON-26	*Homo sapiens*	Grp_VI	LST0091
**L3147**	MHOM/IR/95/YAGHOOBIL2	*L. major*	Iran	M.E	MON-26	*Homo sapiens*	Grp_VI	LST0093
**L3499**	MHOM/ML/97/LSL31	*L. major*	Mali	W.AF	MON-117	*Homo sapiens*	Grp_VI	LST0110
**L3535**	MHOM/CM/97/MOK1	*L. major*	Cameroon	C.AF	MON-26	*Homo sapiens*	Grp_VI	LST0111
**L4063**	MHOM/SN/2000/LSL50	*L. major*	Senegal	W.AF	MON-74	*Homo sapiens*	Grp_VI	LST0125
**L4067**	MHOM/BF/2000/COU5	*L. major*	Burkina Faso	W.AF	MON-74	*Homo sapiens*	Grp_VI	LST0126
**L4148**	MHOM/NE/2001/2001/FRI01	*L. major*	Niger	W.AF	MON-74	*Homo sapiens*	Grp_VI	LST0127
**L4613**	MHOM/SD/2003/LCB33	*L. major*	Sudan	E. AF	MON-74	*Homo sapiens*	Grp_VI	LST0134
**L4797**	MHOM/SN/2004/LCB39	*L. major*	Senegal	W.AF	MON-26	*Homo sapiens*	Grp_VI	LST0136
**L4821**	MHOM/00/2004/LEI24	*L. major*	Burkina Faso	W.AF	MON-26	*Homo sapiens*	Grp_VI	LST0137
**L4886**	MHOM/BF/2004/REN04-8	*L. major*	Burkina Faso	W.AF	MON-26	*Homo sapiens*	Grp_VI	LST0138
**L5622**	MHOM/CF/2008/IPB1	*L. major*	C.A.F	C.AF	MON-305	*Homo sapiens*	Grp_VI	LST0139
**L0075**	MHOM/FR/78/LEM75	*L. infantum*	France	S.E	MON-1	*Homo sapiens*	Grp_VII	LST0001
**L0189**	MHOM/FR/80/LEM189	*L. infantum*	France	S.E	MON-11	*Homo sapiens*	Grp_VII	LST0001
**L0236**	MHOM/IT/79/ISS7	*L. infantum*	Italy	S.E	MON-27	*Homo sapiens*	Grp_VII	LST0001
**L0260**	MHOM/ES/81/LEM260	*L. infantum*	Spain	S.E	MON-34	*Homo sapiens*	Grp_VII	LST0001
**L0307**	MHOM/ES/81/BCN1	*L. infantum*	Spain	S.E	MON-29	*Homo sapiens*	Grp_VII	LST0001
**L0461**	MHOM/DZ/83/LIPA120	*L. infantum*	Algeria	N. AF	MON-34	*Homo sapiens*	Grp_VII	LST0001
**L0538**	MHOM/FR/84/LEM538	*L. infantum*	France	S.E	MON-34	*Homo sapiens*	Grp_VII	LST0001
**L0622**	MHOM/CF/00/CONJUGO	*L. infantum*	C.A.F	C.AF	MON-1	*Homo sapiens*	Grp_VII	LST0001
**L0666**	MNYT/CN/80/RACOONDOG	*L. infantum*	China	E.A	MON-34	*Nyctomys procyonoides*	Grp_VII	LST0001
**L0895**	MHOM/IT/86/ISS218	*L. infantum*	Italy	S.E	MON-72	*Homo sapiens*	Grp_VII	LST0001
**L1208**	MHOM/EG/87/RTC2	*L. infantum*	Egypt	M.E	MON-98	*Homo sapiens*	Grp_VII	LST0001
**L1240**	MHOM/YE/88/LEM1240	*L. infantum*	Yemen	M.E	MON-1	*Homo sapiens*	Grp_VII	LST0001
**L1315**	MCAN/SN/88/LEM1315	*L. infantum*	Senegal	W.AF	MON-1	*Canis familiaris*	Grp_VII	LST0001
**L1355**	IPER/FR/88/LEM1355	*L. infantum*	France	S.E	MON-105	*Homo sapiens*	Grp_VII	LST0001
**L1393**	IPER/FR/88/LEM1393	*L. infantum*	France	S.E	MON-77	*Phlebotomus*	Grp_VII	LST0001

						perniciosus		
**L1424**	MCAN/FR/87/RM1	*L. infantum*	France	S.E	MON-108	*Canis familiaris*	Grp_VII	LST0001
**L1671**	MCAN/DZ/89/LEM1671	*L. infantum*	Algeria	N. AF	MON-77	*Canis familiaris*	Grp_VII	LST0001
**L1674**	MCAN/ES/89/BCN34	*L. infantum*	Spain	S.E	MON-11	*Canis familiaris*	Grp_VII	LST0001
**L2109**	MCAN/SY/90/LEM2109	*L. infantum*	Syria	M.E	MON-1	*Canis familiaris*	Grp_VII	LST0001
**L2205**	MHOM/ES/90/BCN61	*L. infantum*	Spain	S.E	MON-28	*Homo sapiens*	Grp_VII	LST0001
**L2289**	MHOM/GR/90/LA1037	*L. infantum*	Greece	S.E	MON-1	*Homo sapiens*	Grp_VII	LST0001
**L2298**	MHOM/ES/91/LEM2298	*L. infantum*	Spain	S.E	MON-183	*Homo sapiens*	Grp_VII	LST0001
**L2479**	MHOM/FR/88/LPMA74ER	*L. infantum*	France	S.E	MON-186	*Homo sapiens*	Grp_VII	LST0001
**L2629**	MCAN/MA/93/LEM2629	*L. infantum*	Morocco	N. AF	MON-1	*Canis familiaris*	Grp_VII	LST0001
**L2791**	MHOM/IT/93/ISS833	*L. infantum*	Italy	S.E	MON-190	*Homo sapiens*	Grp_VII	LST0001
**L2813**	MHOM/TR/94/LPN101	*L. infantum*	Turkey	M.E	MON-1	*Homo sapiens*	Grp_VII	LST0001
**L2844**	MHOM/PT/94/IMT202	*L. infantum*	Portugal	S.E	MON-29	*Homo sapiens*	Grp_VII	LST0001
**L3161**	MCAN/IR/94/MOHEBL1	*L. infantum*	Iran	M.E	MON-1	*Canis familiaris*	Grp_VII	LST0001
**L3338**	MHOM/ES/97/LGE1	*L. infantum*	Spain	S.E	MON-1	*Homo sapiens*	Grp_VII	LST0001
**L3346**	MHOM/IT/97/LPN149	*L. infantum*	Italy	S.E	MON-1	*Homo sapiens*	Grp_VII	LST0001
**L3357**	MCAN/ /../LV755	*L. infantum*	U.K	W.E	MON-1	*Canis familiaris*	Grp_VII	LST0001
**L3386**	MCAN/IL/97/LRC-L720	*L. infantum*	Israel	M.E	MON-1	*Canis familiaris*	Grp_VII	LST0001
**L3519**	MHOM/PT/98/IMT238	*L. infantum*	Portugal	S.E	MON-80	*Homo sapiens*	Grp_VII	LST0001
**L3546**	MHOM/DL/98/LUB1	*L. infantum*	Germany	W.E	MON-1	*Homo sapiens*	Grp_VII	LST0001
**L3637**	MHOM/ES/97/LLM719	*L. infantum*	Spain	S.E	MON-253	*Homo sapiens*	Grp_VII	LST0001
**L3675**	MHOM/TN/98/20MO	*L. infantum*	Tunisia	N. AF	MON-1	*Homo sapiens*	Grp_VII	LST0001
**L3895**	MCAN/AL/98/C78L574a	*L. infantum*	Albania	S.E	MON-1	*Canis familiaris*	Grp_VII	LST0001
**L3940**	MCAN/PT/99/BOBI	*L. infantum*	Portugal	S.E	MON-98	*Canis familiaris*	Grp_VII	LST0001
**L3962**	MHOM/AL/2000/516LEZH	*L. infantum*	Albania	S.E	MON-1	*Homo sapiens*	Grp_VII	LST0001
**L4224**	MHOM/ES/2000/LLM938	*L. infantum*	Spain	S.E	MON-282	*Homo sapiens*	Grp_VII	LST0001
**L4228**	MHOM/ES/97/LLM615	*L. infantum*	Spain	S.E	MON-285	*Homo sapiens*	Grp_VII	LST0001
**L4610**	MHOM/PT/2001/IMT266B	*L. infantum*	Portugal	S.E	MON-1	*Homo sapiens*	Grp_VII	LST0001
**L4650**	MHOM/FR/2003/LPN221	*L. infantum*	France	S.E	MON-1	*Homo sapiens*	Grp_VII	LST0001
**L4749**	MHOM/FR/2004/LPN228	*L. infantum*	France	S.E	MON-1	*Homo sapiens*	Grp_VII	LST0001
**L4826**	MHOM/FR/2004/LPN236	*L. infantum*	France	S.E	MON-1	*Homo sapiens*	Grp_VII	LST0001
**L4838**	MHOM/FR/2004/LPN235	*L. infantum*	France	S.E	MON-1	*Homo sapiens*	Grp_VII	LST0001
**L4840**	MHOM/FR/2004/LPN237	*L. infantum*	France	S.E	MON-1	*Homo sapiens*	Grp_VII	LST0001
**L5425**	MHOM/IL/2006/LRC-L1296	*L. infantum*	Israel	M.E	MON-1	*Homo sapiens*	Grp_VII	LST0001
**L5426**	MHOM/IL/2006/LRC-L1300	*L. infantum*	Israel	M.E	MON-1	*Homo sapiens*	Grp_VII	LST0001
**L5427**	MHOM/IL/2007/LRC-L1303	*L. infantum*	Israel	M.E	MON-1	*Homo sapiens*	Grp_VII	LST0001
**L5542**	MCAN/UZ/2007/LRC-L1313	*L. infantum*	Uzbekistan	C.A	MON-1	*Canis familiaris*	Grp_VII	LST0001
**L5695**	MCAN/DZ/2008/ENV48	*L. infantum*	Algeria	N. AF	MON-281	*Canis familiaris*	Grp_VII	LST0001

**L0991**	MHOM/LB/84/SALTI1	*L. archibaldi*	Lebanon	M.E	MON-82	*Homo sapiens*	Grp_VII	LST0003
**L0995**	MHOM/LB/84/SALTI3	*L. archibaldi*	Lebanon	M.E	MON-82	*Homo sapiens*	Grp_VII	LST0003
**L1070**	MHOM/IN/00/LRC-L51	*L. donovani*	India	I.S	MON-18	*Homo sapiens*	Grp_VII	LST0003
**L1825**	MHOM/KE/89/EB59	*L. archibaldi*	Kenya	E. AF	MON-82	*Homo sapiens*	Grp_VII	LST0003
**L1859**	MHOM/TN/00/TUNIS	*L. archibaldi*	Tunisia	N. AF	MON-82	*Homo sapiens*	Grp_VII	LST0003
**L2431**	MHOM/PT/92/IMT180	*L. donovani*	Portugal	S.E	MON-18	*Homo sapiens*	Grp_VII	LST0003
**L3588**	MHOM/ES/98/DPPE28	*L. infantum*	Spain	S.E	MON-24	*Homo sapiens*	Grp_VII	LST0004
**L4616**	MHOM/MA/2003/TIM10	*L. infantum*	Morocco	N. AF	MON-29	*Homo sapiens*	Grp_VII	LST0004
**L5110**	MHOM/ES/96/LLM-558	*L. infantum*	Spain	S.E	MON-302	*Homo sapiens*	Grp_VII	LST0004
**L0356**	MHOM/FR/82/LEM356	*L. infantum*	France	S.E	MON-33	*Homo sapiens*	Grp_VII	LST0007
**L2893**	MHOM/ES/88/LLM175	*L. infantum*	Spain	S.E	MON-198	*Homo sapiens*	Grp_VII	LST0007
**L4929**	MHOM/FR/2005/LPN253	*L. infantum*	France	S.E	MON-80	*Homo sapiens*	Grp_VII	LST0007
**L1242**	MHOM/YE/88/LEM1242	*L. donovani*	Yemen	M.E	MON-31	*Homo sapiens*	Grp_VII	LST0010
**L1863**	MHOM/IN/61/L13	*L. donovani*	India	I.S	MON-31	*Homo sapiens*	Grp_VII	LST0010
**L0980**	MHOM/ET/84/ADDIS164	*L. donovani*	Ethiopia	E. AF	MON-83	*Homo sapiens*	Grp_VII	LST0013
**L4272**	MHOM/SD/87/UGX-MARROW	*L. donovani*	Sudan	E. AF	MON-31	*Homo sapiens*	Grp_VII	LST0013
**L0358**	MHOM/IN/54/SC23	*L. donovani*	India	I.S	MON-38	*Homo sapiens*	Grp_VII	LST0014
**L0707**	MHOM/KE/55/LRC-L53	*L. donovani*	Kenya	E. AF	MON-36	*Homo sapiens*	Grp_VII	LST0014
**L2388**	IMRT/ET/90/TESHOME210	*L. donovani*	Ethiopia	E. AF	MON-37	*Phlebotomus martini*	Grp_VII	LST0015
**L4273**	MHOM/KE/73/MRC74	*L. donovani*	Kenya	E. AF	MON-2	*Homo sapiens*	Grp_VII	LST0015
**L0138**	MHOM/IN/00/DEVI	*L. donovani*	India	I.S	MON-2	*Homo sapiens*	Grp_VII	LST0019
**L0232**	MCAN/IT/76/ISS2	*L. archibaldi*	Italy	S.E	MON-82	*Canis familiaris*	Grp_VII	LST0022
**L0417**	MHOM/DZ/82/LIPA59	*L. infantum*	Algeria	N. AF	MON-24	*Homo sapiens*	Grp_VII	LST0024
**L0425**	MHOM/DZ/83/LEM425	*L. infantum*	Algeria	N. AF	MON-80	*Homo sapiens*	Grp_VII	LST0026
**L0494**	MHOM/SD/82/GILANI	*L. infantum*	Sudan	E. AF	MON-30	*Homo sapiens*	Grp_VII	LST0029
**L0659**	MHOM/GR/00/LEM659	*L. infantum*	Greece	S.E	MON-80	*Homo sapiens*	Grp_VII	LST0036
**L0668**	MHOM/CN/00/WANGJIE-1	*L. donovani*	China	E.A	MON-35	*Homo sapiens*	Grp_VII	LST0037
**L0698**	MHOM/ET/67/HU3	*L. donovani*	Ethiopia	E. AF	MON-18	*Homo sapiens*	Grp_VII	LST0038
**L0719**	IMRT/KE/62/LRC-L57	*L. donovani*	Kenya	E. AF	MON-37	*Phlebotomus martini*	Grp_VII	LST0039
**L0890**	MHOM/SD/66/L46	*L. infantum*	Sudan	E. AF	MON-30	*Homo sapiens*	Grp_VII	LST0040
**L0935**	MCAN/ES/86/LEM935	*L. infantum*	Spain	S.E	MON-77	*Homo sapiens*	Grp_VII	LST0043
**L0946**	MHOM/SU/84/MARZ-KRIM	*L. donovani*	Ukraine	E.E	MON-73	*Homo sapiens*	Grp_VII	LST0044
**L1005**	MHOM/ET/72/GEBRE1	*L. archibaldi*	Ethiopia	E. AF	MON-82	*Homo sapiens*	Grp_VII	LST0046
**L1014**	MHOM/ET/84/ADDIS135	*L. donovani*	Ethiopia	E. AF	MON-32	*Homo sapiens*	Grp_VII	LST0047
**L1019**	MHOM/ET/82/BEKELE	*L. donovani*	Ethiopia	E. AF	MON-31	*Homo sapiens*	Grp_VII	LST0049
**L1043**	MHOM/MT/85/BUCK	*L. infantum*	Malta	S.E	MON-78	*Homo sapiens*	Grp_VII	LST0050
**L1153**	MHOM/SD/62/3S	*L. infantum*	Sudan	E. AF	MON-81	*Homo sapiens*	Grp_VII	LST0055
**L1154**	MHOM/SD/00/DH177	*L. infantum*	Sudan	E. AF	MON-81	*Homo sapiens*	Grp_VII	LST0056

**L1560**	MHOM/ES/89/BCN32	*L. infantum*	Spain	S.E	MON-33	*Homo sapiens*	Grp_VII	LST0062
**L1733**	MHOM/IT/85/ISS175	*L. infantum*	Italy	S.E	MON-111	*Homo sapiens*	Grp_VII	LST0066
**L1764**	IARI/PT/89/IMT171	*L. infantum*	Portugal	S.E	MON-24	*Phlebotomus ariasi*	Grp_VII	LST0067
**L2141**	IALE/CN/88/Turfan10	*L. donovani*	China	E.A	MON-138	*Phlebotomus alexandri*	Grp_VII	LST0072
**L2180**	MHOM/IT/90/ISS510	*L. infantum*	Italy	S.E	MON-136	*Homo sapiens*	Grp_VII	LST0073
**L2455**	MHOM/MA/92/LEM2455	*L. infantum*	Morocco	N. AF	MON-24	*Homo sapiens*	Grp_VII	LST0078
**L2481**	MHOM/IT/91/ISS666	*L. infantum*	Italy	S.E	MON-185	*Homo sapiens*	Grp_VII	LST0079
**L2506**	MHOM/DZ/92/CRE21	*L. infantum*	Algeria	N. AF	MON-78	*Homo sapiens*	Grp_VII	LST0080
**L2677**	MHOM/YE/93/LEM2677	*L. donovani*	Yemen	M.E	MON-191	*Homo sapiens*	Grp_VII	LST0081
**L2793**	MHOM/IT/93/ISS800	*L. infantum*	Italy	S.E	MON-188	*Homo sapiens*	Grp_VII	LST0082
**L2794**	MHOM/IT/93/ISS822	*L. infantum*	Italy	S.E	MON-201	*Homo sapiens*	Grp_VII	LST0083
**L2795**	MHOM/IT/92/ISS683	*L. infantum*	Italy	S.E	MON-187	*Homo sapiens*	Grp_VII	LST0084
**L2884**	MHOM/DZ/94/CRE64	*L. infantum*	Algeria	N. AF	MON-33	*Homo sapiens*	Grp_VII	LST0086
**L2894**	MHOM/ES/92/LLM373	*L. infantum*	Spain	S.E	MON-199	*Homo sapiens*	Grp_VII	LST0088
**L2975**	MHOM/MA/95/CRE72	*L. donovani*	Morocco	N. AF	MON-37	*Homo sapiens*	Grp_VII	LST0089
**L3079**	MHOM/SA/91/WR1063	*L. donovani*	Saudi Arabia	M.E	MON-18	*Homo sapiens*	Grp_VII	LST0090
**L3141**	MHOM/FR/95/LEM3141	*L. infantum*	France	S.E	MON-28	*Homo sapiens*	Grp_VII	LST0092
**L3437**	MHOM/IT/95/GIIPcos	*L. infantum*	Italy	S.E	MON-29	*Homo sapiens*	Grp_VII	LST0107
**L3443**	MHOM/SD/97/LEM3443	*L. archibaldi*	Sudan	E. AF	MON-257	*Homo sapiens*	Grp_VII	LST0108
**L3463**	MHOM/SD/97/LEM3463	*L. archibaldi*	Sudan	E. AF	MON-258	*Homo sapiens*	Grp_VII	LST0109
**L3538**	MHOM/FR/97/LPM 178a	*L. infantum*	France	S.E	MON-30	*Homo sapiens*	Grp_VII	LST0112
**L3787**	MCAN/SD/99/LEM3787	*L. archibaldi*	Sudan	E. AF	MON-82	*Canis familiaris*	Grp_VII	LST0113
**L3793**	MHOM/SD/99/LEM3793	*L. donovani*	Sudan	E. AF	MON-276	*Homo sapiens*	Grp_VII	LST0114
**L3941**	MCAN/SD/2000/LEM3941	*L. infantum*	Sudan	E. AF	MON-278	*Canis familiaris*	Grp_VII	LST0116
**L3942**	MCAN/SD/2000/LEM3942	*L. donovani*	Sudan	E. AF	MON-277	*Canis familiaris*	Grp_VII	LST0117
**L3968**	MHOM/TN/99/105 S	*L. infantum*	Tunisia	N. AF	MON-80	*Homo sapiens*	Grp_VII	LST0119
**L3976**	MHOM/PT/2000/IMT261	*L. infantum*	Portugal	S.E	MON-1	*Homo sapiens*	Grp_VII	LST0120
**L3988**	MCAN/SD/2000/LEM3988	*L. infantum*	Sudan	E. AF	MON-267	*Canis familiaris*	Grp_VII	LST0122
**L4043**	MHOM/IL/79/LANSBERG	*L. donovani*	Israel	M.E	MON-280	*Homo sapiens*	Grp_VII	LST0123
**L4046**	MHOM/IL/87/BENAMI	*L. donovani*	Israel	M.E	MON-37	*Homo sapiens*	Grp_VII	LST0124
**L4223**	MHOM/ES/95/LLM480	*L. infantum*	Spain	S.E	MON-228	*Homo sapiens*	Grp_VII	LST0128
**L4227**	MHOM/ES/99/LLM855	*L. infantum*	Spain	S.E	MON-284	*Homo sapiens*	Grp_VII	LST0129
**L4319**	MHOM/SD/2001/AHSAF11	*L. donovani*	Sudan	E. AF	MON-18	*Homo sapiens*	Grp_VII	LST0130
**L4392**	MHOM/TN/2002/LC38	*L. infantum*	Tunisia	N. AF	MON-24	*Homo sapiens*	Grp_VII	LST0131
**L4474**	MHOM/LK/2002/L80	*L. donovani*	Sri Lanka	I.S	MON-37	*Homo sapiens*	Grp_VII	LST0132
**L4537**	MHOM/IN/2003/LEM4537	*L. donovani*	India	I.S	MON-37	*Homo sapiens*	Grp_VII	LST0133
**L4701**	MHOM/GR/2003/GH15	*L. infantum*	Greece	S.E	MON-98	*Homo sapiens*	Grp_VII	LST0135
**L5751**	MHOM/TR/2005/CUK1	*L. donovani*	Turkey	M.E	MON-309	*Homo sapiens*	Grp_VII	LST0140

**L2208**	MHOM/BR/81/M6426	*L. lainsoni*	Brazil	S.A	MON-149	*Homo sapiens*	ND	ND
**L2700**	MHOM/BO/90/AN	*L. braziliensis*	Bolivia	S.A	MON-214	*Homo sapiens*	ND	ND
**L4762**	MHOM/GF/2004/LAV014	*L. guyanensis*	French Guiana	S.A	MON-45	*Homo sapiens*	ND	ND

C.A.F, Central African Republic; U.K, United Kingdom.

C.A, Central Asia; C.AF, Central Africa; E.A, Eastern Asia; E.AF, Eastern Africa; E.E, Eastern Europe; I.S, Indian Subcontinent; M.E, Middle East; N.AF, Northern Africa; S.E, Southern Europe; W.A, Western Asia; W.AF, Western Africa; W.E, Western Europe, S.A, Southern America.

LST: *Leishmania* sequence type

ND: Not determined
