# Supplementary material for: Genetic Structure and Evolution of the Leishmania Genus in Africa and Eurasia: What Does MLSA Tell Us
Source: PLoS Negl Trop Dis. 2013 Jun 13;7(6):e2255. doi: 10.1371/journal.pntd.0002255 (PMC3681676; doi:10.1371/journal.pntd.0002255)
Supplement: Table S2 — Statistical assessment of the congruence between the Maximum Likelihood (ML) and Bayesian tree topologies. a: Log Likelihood of the ML and Bayesian tree topologies based on the concatenated nucleotides sequences b: differences in Log likelihood between the ML and Bayesian trees. The congruence between ML and Bayesian tree topologies was confirmed with the SH test (p-value = 0.65). (PDF) [file pntd.0002255.s010.pdf]

Table S2. Statistical assessment of the congruence between the Maximum Likelihood (ML) and Bayesian tree topologies.

**Tree of the concatenated nucleotides**	**-ln L [a]**	**Diff -ln L [b]**	***p*-value**
ML tree topology	29555.84144		
Bayesian tree topology	29607.08844	51.24700	0.65
