# Supplementary material for: Genetic Structure and Evolution of the Leishmania Genus in Africa and Eurasia: What Does MLSA Tell Us
Source: PLoS Negl Trop Dis. 2013 Jun 13;7(6):e2255. doi: 10.1371/journal.pntd.0002255 (PMC3681676; doi:10.1371/journal.pntd.0002255)
Supplement: Table S3 — Statistical assessment of the congruence between the concatenated nucleotide and amino acid tree topologies. a: Log Likelihood of the ML tree topologies of the concatenated nucleotide sequences (concatenated nt) and the concatenated amino acid sequences (concatenated AA). b: differences in Log likelihood between nucleotide and amino acid trees. The SH test indicated no significant differences in Log likelihood (p-value = 0.36). (PDF) [file pntd.0002255.s011.pdf]

Table S3. Statistical assessment of the congruence between the concatenated nucleotide and amino acid tree topologies.

**ML tree topology**	**-ln L [a]**	**Diff -ln L [b]**	***p*-value**
Concatenated nt	29607.08844		
Concatenated AA	30548.32436	941.23592	0.36
