# Supplementary material for: Genetic Structure and Evolution of the Leishmania Genus in Africa and Eurasia: What Does MLSA Tell Us
Source: PLoS Negl Trop Dis. 2013 Jun 13;7(6):e2255. doi: 10.1371/journal.pntd.0002255 (PMC3681676; doi:10.1371/journal.pntd.0002255)
Supplement: Table S4 — Statistical assessment of the congruence between the MLSA and MLEE datasets. a: Log Likelihood of the MLSA and MLEE NJ tree topologies b: differences in Log likelihood between MLSA and MLEE NJ trees. The overall congruence was assessed by comparison of the likelihood between the concatenated nucleotide (nt) Neighbor Joining (NJ) tree (MLSA) and the isoenzymatic data (MLEE). The SH test showed significant congruence between the MLSA and MLEE NJ trees (p-value = 0.16), indicating that the seven clusters were conserved with both approaches. (PDF) [file pntd.0002255.s012.pdf]

Table S4. Statistical assessment of the congruence between the MLSA and MLEE datasets.

**NJ tree topology**	**-ln L [a]**	**Diff -ln L [b]**	***P*-value**
Concatenated nt (MLSA)	22989.74334	(best)	
Iso-enzymatic data (MLEE)	25030.88926	2041.14592	**0.16**
