# Supplementary material for: Genetic Structure and Evolution of the Leishmania Genus in Africa and Eurasia: What Does MLSA Tell Us
Source: PLoS Negl Trop Dis. 2013 Jun 13;7(6):e2255. doi: 10.1371/journal.pntd.0002255 (PMC3681676; doi:10.1371/journal.pntd.0002255)
Supplement: Table S5 — Statistical assessment of the congruence between individual MLSA clusters and MLEE groups. a: Log Likelihood of the individual NJ trees for the MLSA clusters and MLEE groups (only clusters I, II, VI and VII were analyzed). b: pairwise differences in Log likelihood between individual NJ trees for the MLSA clusters and MLEE groups. Only cluster II showed significant congruence between the MLSA and MLEE approaches, as indicated by the SH test (p-value = 0.28). (PDF) [file pntd.0002255.s013.pdf]

Table S5. Statistical assessment of the congruence between individual MLSA clusters and MLEE groups.

**NJ tree topology**	**-ln L [a]**	**Diff -ln L [b]**	***P*-value**
Group I MLSA	13355.48940		
Group I MLEE	13491.05094	135.56154	0.00*
Group II MLSA	14714.18758		
Group II MLEE	14806.36916	1184.11436	**0.28**
Group VI MLSA	13399.80026		
Group VI MLEE	13610.39187	210.59161	0.00*
Group VII MLSA	14725.60965		
Group VII MLEE	15324.29407	598.68442	0.00*

* $P < 0.05$
